# Nutrient Intake and Nutrition Status in Vegetarians and Vegans in Comparison to Omnivores - the Nutritional Evaluation (NuEva) Study

**DOI:** 10.3389/fnut.2022.819106

**Published:** 2022-05-16

**Authors:** Christine Dawczynski, Thomas Weidauer, Cora Richert, Peter Schlattmann, Kristin Dawczynski, Michael Kiehntopf

**Affiliations:** ^1^Junior Research Group Nutritional Concepts, Institute of Nutritional Sciences, Friedrich Schiller University, Jena, Germany; ^2^Competence Cluster for Nutrition and Cardiovascular Health (nutriCARD), Halle-Jena-Leipzig, Leipzig, Germany; ^3^Institute of Clinical Chemistry and Laboratory Diagnostics, University Hospital, Jena, Germany; ^4^Department of Medical Statistics, Informatics and Data Science, University Hospital, Jena, Germany; ^5^Department for Pediatrics and Adolescent Medicine, Sophien- and Hufeland Hospital, Weimar, Germany

**Keywords:** vegans, vegetarians, omnivores, nutrient intake, blood lipids, body weight

## Abstract

**Introduction:**

In recent years, vegetarian and vegan diets became increasingly important as they are associated with beneficial health outcomes. Therefore, the NuEva study compares the impact of flexitarian, vegetarian, or vegan diets with omnivorous nutritional habits on nutrient intake and risk factors for non-communicable diseases.

**Methods:**

A dietary protocol was kept over five days and blood and 24h urine samples were collected to examine the impact of dietary habits [omnivores, *n* = 65 (Median/Interquartile range: 33/17 yrs.), flexitarians, *n* = 70 (30/17 yrs.), ovo-lacto vegetarians, *n* = 65 (28/14 yrs.), vegans, *n* = 58 (25/10 yrs.)] on nutrient intake, nutrient concentrations in plasma, serum or 24h urine, body composition, and blood lipids.

**Results:**

The increased exclusion of animal based foods in the diet (omnivores < flexitarians < vegetarians < vegans) is associated with a decreased intake of energy, saturated fat, cholesterol, disaccharides, and total sugar as well an increased intake of dietary fibers, beta carotene, vitamin E and K. The combined index of the B12 status (4cB12 score) in vegetarians (0.02/0.75) was lower compared to omnivores (0.34/0.58; *p* ≤ 0.05) and flexitarians (0.24/0.52; *p* ≤ 0.05). In omnivores vitamin A, vitamin E, ferritin, and the urinary excretion of selenium, iodine, and zinc were higher than in vegans (*p* ≤ 0.05). In contrast, vegans had the highest concentrations of biotin, folate, and vitamin C. Flexitarians, vegetarians, and vegans had a lower body weight, BMI, and body fat percentage in comparison to omnivores (*p* ≤ 0.05). In omnivores the concentrations on total cholesterol, total cholesterol/HDL cholesterol ratio, LDL cholesterol, LDL cholesterol/HDL cholesterol ratio, apolipoprotein B, and apolipoprotein B/ apolipoprotein A1 ratio were higher than in vegetarians and vegans (*p* ≤ 0.05).

**Conclusion:**

The NuEva study confirms the position of the Academy of Nutrition and Dietetics that adequately planned vegetarian diets are healthy, nutritionally adequate, and may provide health benefits in the prevention and treatment of non-communicable diseases. Nevertheless, critical nutrients were identified for all groups studied. This highlights the need to develop individual nutritional concepts to ensure an adequate nutrient intake.

## Introduction

Cardiovascular diseases (CVD) are one of the leading health problems worldwide and the main cause of death in German and the European region (1). Up to 40% of cardiovascular disease can be avoided by changing to a healthier diet (2). In recent years, vegetarian and vegan diets became increasingly important as they are associated with beneficial effects on blood lipid profile and a reduced risk of cardiovascular diseases (3, 4). On the other side, vegetarian and vegan diets typically avoid such foods as meat, sausage, fish (vegetarians) and eggs, dairy products, and honey (vegans) which bear the risk of undersupply of essential nutrients such as long-chain n-3 fatty acids (n-3 LC-PUFA), vitamin B2, vitamin B12, vitamin D, calcium, potassium, selenium, and zinc (5). In addition, the availability of ultra-processed meat and dairy alternatives on the supermarkets is growing (6, 7). Vegetarians and vegans consumed more ultra-processed foods such as industrial plant-based meat and dairy substitutes than omnivores. A higher consumption of ultra-processed foods is associated with higher risks of cardiovascular, coronary heart, and cerebrovascular diseases (8).

The predominant dietary pattern in Germany is characterized by high intake of foods of animal origin whereas the consumption of foods of plant origin is comparable low (9). This dietary pattern results in a low intake of dietary fibers, polyunsaturated fatty acids (PUFA), and secondary plant compounds which is associated with an increase in cardiovascular risk factors such as blood lipids (10–12).

The hype surrounding the vegetarian and vegan diet and the high prevalence of the omnivorous dietary pattern in combination with the likelihood for over- and undersupply of nutrients following the adoption of these eating habits highlights the need of extensive data collection to develop evidence-based recommendations.

In this context, a central objective of the NuEva study is to assess nutrient intake in the studied diets and compare of nutrient concentrations in plasma, serum and 24 h urine as well as the impact on cardiovascular risk factors.

## Materials and Methods

### Study Design (Screening)

In summer/autumn 2018, healthy women and men between 18 to <70 years were recruited by press release and flier. The flier was distributed on the universities in Jena, Halle and Leipzig and in refectories, cafeterias, fitness studios, youth clubs, restaurants and coffee shops. Interested individuals initially complete a telephone pre-screening. As precondition, one of the four diets studied had been implemented since at least 1 year before enrollment. The adherence to one of the four diets (omnivores, flexitarians, vegetarians, vegans) was assessed by a self-created questionnaire and a dietary protocol over 5d. The study protocol listed the following exclusion criteria (13):

- Patients with diseases of the parathyroid, diseases necessitating regular phlebotomies, or patients with acute or chronic disease, which could affect the results of the present study. In addition, the following treatments precluding participation (at least 3 months prior to study start) resulted in an exclusion from the NuEva study
- Weight loss or weight gain (> 3 kg).
- Fundamental changes in dietary habits.
- Hormone replacement therapy.
- Elite athletes (>15 h of strenuous physical activity per week).
- Pregnancy or lactation.

**Figure 1:**
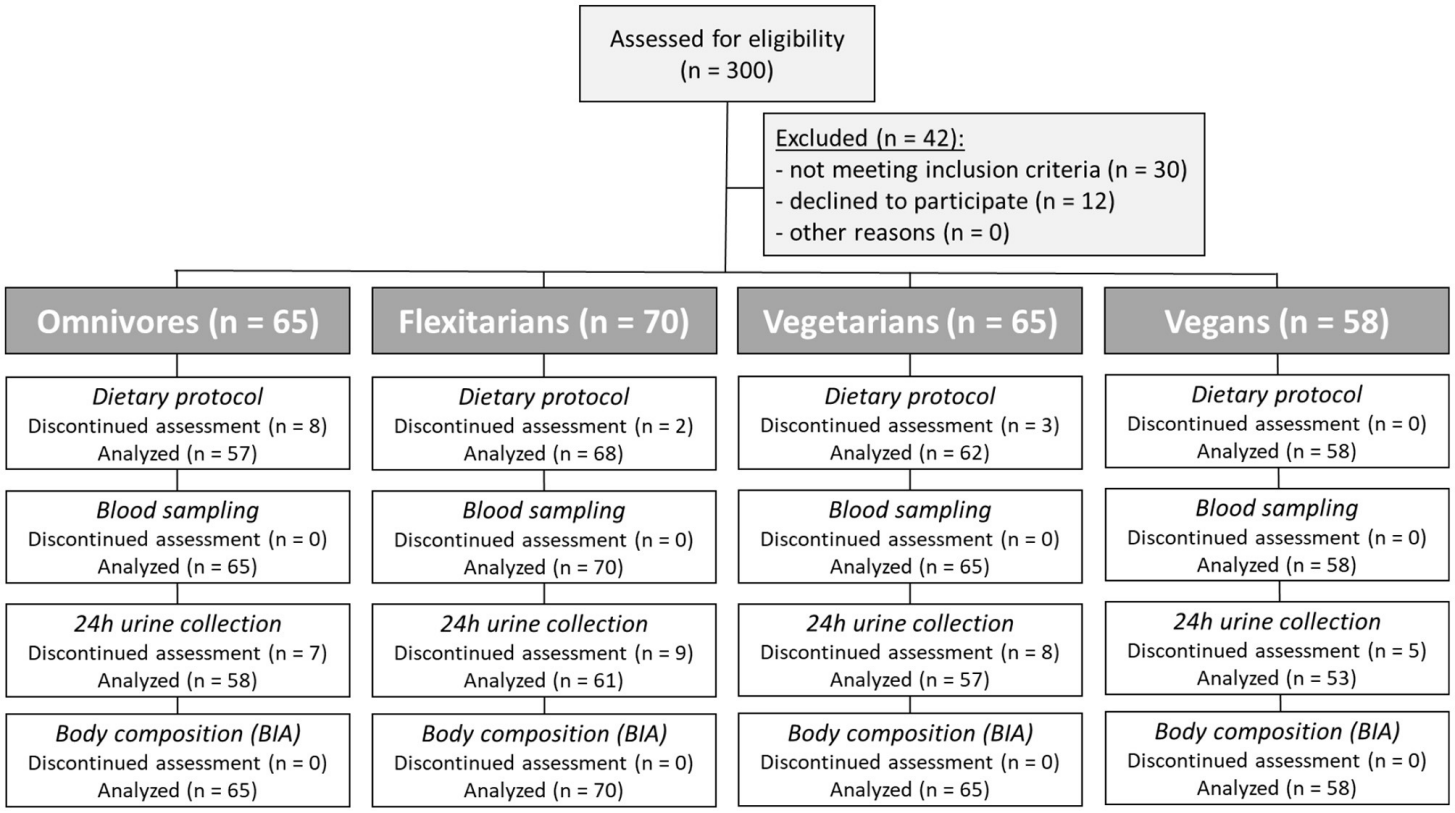
Flow diagram of the participants. Three hundred subjects were enrolled in this study. Forty-two subjects were excluded since they did not meet the inclusion criteria or declined to participate. Based on their eating habits in advance, the participants were divided into four groups (omnivores, flexitarians, vegetarians, and vegans).

Following the informed consent and confirmation of the in- and exclusion criteria, participants are scheduled for the baseline assessment. In total, 65 omnivores (daily consumption of meat and sausage, inclusive chicken/poultry, beef, pork etc.; consumption of fish), 70 flexitarians (occasional consumption of meat and sausage, inclusive chicken/poultry, beef, pork etc. (pre-dominantly high-quality products, ≤ two times/week); consumption of fish), 65 ovo-lacto vegetarians (no consumption of meat, sausage, fish), and 58 vegans (no consumption of foods of animal origin) participate on the NuEva screening (Table 1; Figure 1). The diet groups ovo-lacto vegetarians and vegans are partially summarized under the term vegetarian/vegan diets in the further course of the manuscript.

**Table 1:** Characteristics of the study collective - NuEva-screening [Median/Interquartile range (IQR); (Min - Max)].

	**Group 1** **40 w, 25 m**				**Group 2** **56 w, 14 m**				**Group 3** **47 w, 18 m**				**Group 4** **41 w, 17 m**			
Age (years)	33.0	/	17.0	a	29.5	/	16.8	a	28.0	/	14.0	a,b	25.0	/	9.8	b
	(18–61)				(19–69)				(18–65)				(19–56)			
Implementation of	32.0	/	20.0	a	8.0	/	17.8	b	6.0	/	10.0	b	3.0	/	3.0	c
the diet (years)	(1–61)				(1–68)				(1–34)				(1–34)			

Groups: 1 = omnivores, 2 = flexitarians, 3 = vegetarians, 4 = vegans. *Diet groups with different indices differ significantly (p < 0.05).

To record and document the variety in dietary practices within and between the groups, the run-in phase of the NuEva study included full self-reporting of individual dietary intake over 5 days. The dietary record based on the template “Freiburger Ernährungsprotokoll” which was provided by PRODI^®^ version 6.4 (Nutri-Science, Stuttgart, Germany) and includes common foods and usual portion sizes. The template was adapted on the NuEva study by adding foods which are favored in vegetarian and vegan diets such as tofu, vegan yogurt alternatives and plant drinks, soy products, seitan, tempeh, maple and agave syrup (further foods could be added individually). Foods which were basically not contained in PRODI^®^ were created and the nutritional information was taken from the packaging (inclusive fortification with e.g., vitamin B12, calcium). The daily energy and nutrient intake was calculated by the software package PRODI^®^. The nutrient intake from supplements was not considered in the calculation of nutrient intake by the dietary protocols (reason: irregular intake and great variety of the supplements, no information on nutrient bioavailability from the used supplements).

Available questionnaires from the German National Consumption Survey II (NVS II) and the German health interview and examination survey for adults (DEGS1) are used to consider the socio-economic status as a confounding factor (14). In detail, the questionnaires include a set of questions about marital status, household size, educational achievement, income, and occupation as well as employment status. In addition to this, participants filled out questionnaires to assess physical activity (15), and health and disease status (inclusive medication use).

Blood was taken by venipuncture between 7:30 AM and 10:30 AM after at least 12 h overnight fast. The urine was collected over 24h directly before phlebotomy. The collection began after the morning urine on the day before. Afterward the urine was completely collected in a special container for the next 24 h. After the morning urine on day 2 (day of phlebotomy) the collection was finished. The total volume was documented, and eight Sarstedt^®^ tubes á 9 ml were filled by the participants according to a standardized operating procedure. The aliquots were stored on a cool place and had to be hand at the study center until 10:00 am. Here the aliquots were frozen immediately (−20°C). Body weight, height, and waist circumferences were measured, respectively, by the same trained study nurse to the nearest half-kilogram or half-centimeter, with patients wearing light clothing with bare feet (one measurement). Waist circumference was measured midway between the lower rib margin and the iliac crest (a thumb's breadth above the navel). For measurement calibrated instruments were used (scale with integrated stadiometer: seca813, Hamburg, Germany; ergonomic tape measure: seca212, Hamburg, Germany).

Body composition was assessed by Body Impedance Analyzer [Data Input, Germany; exactness of measurement: 0.5 % of measurement value (Reactance)/± 2.0 % of measurement value (Resistance)]. The study protocol was reviewed and approved by the Ethical Committee of the Friedrich-Schiller-University of Jena (number: 5504-03/18). The NuEva study was registered before launching (Clinical-Trials.gov Identifier: NCT03582020).

### Sample Collection and Biochemical Analyses

Fasting peripheral venous blood samples were collected and centrifuged (10 min, 2,762 g, 4°C) for separation of plasma and serum. The 24h urine was aliquoted. Study parameters were analyzed immediately after blood sampling or urine collection or by using aliquots from serum, plasma, and 24h urine collections which were stored at −20°C (24h urine) or −80°C (serum, plasma) until analysis. The samples were prepared according to standard operation procedures. The analyses of biotin, methylmalonic acid, vitamin B2, vitamin C, and iodine were performed by Dianovis GmbH (Supplementary Table S1). Further chemical parameters in serum, plasma and urine were measured by using an Abbott Architect CI 16200 analyzer or HPLC according to the manufacturer's recommendations (Supplementary Table S1). Blood count was analyzed by XN 1000 (Sysmex^®^). Selenium and zinc were quantified by atomic absorption spectroscopy (Supplementary Table S1).

### Statistical Methods

Statistical analyses were performed using the statistical software 'R‘ (version R i386 3.5.2). The same procedures were used for all studied diet groups. If the data of the four groups follow a normal distribution (tested with Shapiro-Wilk), one-way ANOVA was applied and the differences between specific groups were investigated using pairwise comparison with a two-sample *t-*test (using Benjamini-Hochberg correction). Otherwise, Kruskal-Wallis test with pairwise comparisons using Wilcoxon signed-rank tests (using Benjamini-Hochberg correction) were used.

The same analysis was performed for men and women subgroups and adjusted data sets as well. A standard ANCOVA was applied to the data to detect if a covariate (age, BMI, sex) influenced a specific variable. This influence meant that the correlation of the variable and the covariate was similar in all four groups, large enough (correlation coefficient larger than 0.3 or lower than −0.3) and significant. If the conditions were met, the values of that variable were adjusted for age (i.e., all values were adjusted as if the participants were all 30 years old) or BMI (i.e., all participants had the same BMI of 22). If sex had a significant influence, the statistical analysis was performed for men and women separately. All tests in this section were evaluated with α = 0.05.

The power calculation was conducted for LDL cholesterol/ HDL cholesterol ratio. The calculation based on data by Li et al. (16). A sample size of 44 participants per group had 80% power. We assume a drop-out rate of 25%. Thus, we enrolled at least 55 participants per group. The other parameters are examined exploratively. The power calculation for the NuEva study was conducted with G^*^Power 3.1.9.2 as described in (13). The details on study design, power-calculation, recruitment procedures, study assessments, and intervention protocol have been published previously (13).

## Results

The NuEva participants' age ranged between 18 and 69 years and the collective consisted of 70% women and 30% men (Table 1). In men, the age did not differ significantly between the four studied diet groups. The omnivorous women were slightly older than the women in the other three groups (*p* ≤ 0.05). In the NuEva study population, the higher age in omnivores and flexitarians differed significantly from the lower age in the vegan group (*p* ≤ 0.05; Table 1). The data were adjusted for age and for BMI, if a significant influence was observed (marked in Tables 2–**6**).

**Table 2:** Daily intake of energy and macronutrients (self-reports, 5 days)–NuEva-screening [Median/IQR; (Min–Max)].

		**Group 1**				**Group 2**				**Group 3**				**Group 4**			
	**Sex**	**Median**	**/**	**IQR**	**p**	**Median**	**/**	**IQR**	**p**	**Median**	**/**	**IQR**	**p**	**Median**	**/**	**IQR**	**p**
**Energy (kcal)**	All	2,325	/	902	a	2,114	/	789	a	2,088	/	875	a,b	1,829	/	573	b
Men (2,100–3,100)/ Women (1,700–2,500)^§^		(898–5,526)				(1,123–4,499)				(1,029–4,155)				(977–4,169)			
**Carbohydrates** (%)	All	42.2	/	11.7	a	47.2	/	8.1	b	49.2	/	9.3	b	55.1	/	9.2	c
>50% of total energy^§^		(23.5–54.2)				(2.4–63.4)				(16.8–65.9)				(30.8–70.1)			
**Total dietary fiber** (g)	All	24.4	/	13.2	a	27.0	/	10.9	a	30.3	/	15.2	b	36.8	/	10.7	c
30 g/day^§^		(9.5–54.6)				(12.5–63.4)				(6.4–85.0)				(14.8–115.5)			
**Dietary fiber**	All	7.9	/	4.0	a	8.2	/	3.8	a	9.0	/	4.6	a	10.1	/	4.6	b
(water-soluble) (g)		(3.3–17.7)				(3.3–24.7)				(1.8–24.6)				(1.8–30.8)			
**Dietary fiber**	All	16.0	/	8.3	a	16.4	/	6.6	a,b	17.8	/	9.5	a,b	22.0	/	8.8	c
(not water-soluble) (g)		(6.0–38.0)				(7.3–40.0)				(3.7–63.2)				(9.2–79.2)			
**Protein (%)**	All	16.5	/	4.4	a	14.0	/	4.2	b	13.3	/	2.9	c	12.8	/	2.8	c
Approx. 15% of total energy^§^		(9.5–27.8)				(9.8–24.6)				(9.4–23.5)				(8.8–23.0)			
**Fat (%)**	All	35.5	/	10.5	a	32.3	/	6.9	b	33.0	/	7.9	b	26.6	/	7.3	c
Approx. 30% of total energy^§^		(19.9–52.9)				(18.2–47.4)				(18.1–55.8)				(14.3–49.3)			
**Σ** **saturated fatty acids (%)**	All	15.3	/	4.4	a	12.9	/	4.2	b	11.8	/	3.6	b	6.2	/	3.3	c
<10% of total energy^§^		(6.6–23.8)				(4.9–20.6)				(4.3–20.8)				(2.9–14.7)			
**Σ** **monounsaturated fatty acids (%)**	All	11.2	/	4.2	a	9.2	/	2.6	b	9.6	/	4.6	b	8.5	/	3.9	c
≥10% of total energy^§^		(5.9–21.0)				(3.9–20.5)				(3.1–25.1)				(3.2–16.6)			
**Σ** **polyunsaturated fatty acids (%)**	All	4.3	/	1.8	a	4.2	/	2.0	a	5.0	/	2.9	b	6.3	/	2.7	c
≥10% of total energy^§^		(2.0–8.3)				(1.5–11.6)				(1.5–14.7)				(1.3–17.3)			
**Oleic acid (g)**	All	27.6	/	17.4	a	18.8	/	11.7	b	18.7	/	12.1	b	15.8	/	9.0	c
		(7.2–81.6)				(7.8–54.3)				(5.0–94.2)				(3.9–40.8)			
**Palmitic acid (g)**	All	18.9	/	10.3	a	13.2	/	8.0	b	12.1	/	6.9	b	5.3	/	3.3	c
		(7.3–46.1)				(4.7−32.9)				(2.5–24.9)				(1.6–18.4)			
**Stearic acid (g)**	All	8.1	/	6.0	a	5.3	/	3.5	b	4.4	/	2.8	c	1.6	/	1.1	d
		(2.3–23.6)				(1.1–15.9)				(0.8–17.7)				(0.4–9.1)			
**Alpha linolenic acid, ALA (g)**	All	1.5	/	1.2	a	1.2	/	0.8	a	1.3	/	0.9	a	1.4	/	1.1	a
		(0.3–7.1)				(0.5–10.5)				(0.4–7.7)				(0.3–7.7)			
**Linoleic acid, LA (g)**	All	8.4	/	5.3	a	8.0	/	5.4	a	9.2	/	8.0	a	9.9	/	5.7	a
		(1.6–33.3)				(2.0–18.2)				(2.3–36.9)				(2.1–20.7)			
**Arachidonic acid, ARA (g)**	All	0.21	/	0.25	a	0.11	/	0.15	b	0.04	/	0.04	c	0.02	/	0.02	d
		(0.04–1.11)				(0.01–0.47)				(0.01–0.17)				(0.01–0.78)			
**Eicosapentaenoic acid, EPA (g)**	All	0.08	/	0.18	a	0.04	/	0.07	b	0.01	/	0.34	c	0.00	/	0.01	d
		(0.01–1.50)				(0.01–0.61)				(0–1.17)				(0–0.01)			
**Docosapentaenoic acid, DPA (g)**	All	0.05	/	0.09	a	0.04	/	0.05	a	0.03	/	0.05	b	0.00	/	0.01	c
		(0.01–0.42)				(0.00–0.27)				(0–0.44)				(0–0.42)			
**Docosahexaenoic acid, DHA (g)**	All	0.16	/	0.29	a	0.09	/	0.13	b	0.04	/	0.05	c	0.01	/	0.01	d
		(0.01–1.60)				(0.01–0.99)				(0.01–1.90)				(0–0.43)			
**Cholesterol (mg)**	All	395.4	/	193.4	a	223.4	/	194.6	b	131.3	/	136.8	c	28.8	/	42.6	d
<300 mg^§^		(79–1,010)				(0–605)				(16–471)				(0–331)			

*Groups: 1 = omnivores, 2 = flexitarians, 3 = vegetarians, 4 = vegans. Adjusted for age: Σ monounsaturated fatty acids (%)*.

§ *Reference intake: (17)*.

^*^Diet groups with different indices differ significantly (p < 0.05).

Generally, the omnivorous diet is practiced since birth or childhood whereas the flexitarian, vegetarian, and vegan diets were practiced on average for 13, 10, and 4 years (Table 1).

### Socio-Economic Data

Marital and educational status differed significantly between the groups (Supplementary Table S2a). The participants of group 1 to 3 were married or living together with a partner while most vegans were single (*p* ≤ 0.05). Most participants in all groups had a university entrance qualification, whereas the proportion with a secondary school leaving certificate was higher in omnivores. In omnivores and flexitarians, the number of subjects with a completed vocational training was higher than in the vegetarian/vegan groups (*p* ≤ 0.05; Supplementary Table S2a). The NuEva study population's household size varied in size ranging from 1 to 4 without differences between the groups under consideration. In omnivores and flexitarians, the proportion of participants with a household net income > 3000 Euro per month was higher than in the vegetarian/vegan groups. On the other hand, the part with a household net income between 501–800 Euro per month was higher in vegetarians and vegans (*p* ≤ 0.001). The intake of nutritional supplements was comparably high in vegans and low in omnivores (*p* ≤ 0.001; Supplementary Table S2b). The regular intake of vitamin B12 and iron supplements was higher in the vegans than in the other groups studied (*p* ≤ 0.05).

### Nutrient Intake

The energy intake varied between the four studied groups with the highest intakes in both the omnivores and flexitarians and the lowest intake in vegans (*p* ≤ 0.01). The intake of carbohydrates, dietary fibers, protein, and fat also varied with substantial differences between the omnivores and the vegetarian/vegan diets (*p* ≤ 0.05). The intake of carbohydrates and dietary fibers increased in the following order: omnivores < flexitarians < vegetarians < vegans, the intake of protein and fat decreased in parallel (Table 2). The consumed dietary fibers consisted of approx. 30% water-soluble fibers and approx. 70% non-water-soluble fibers (Table 2). In vegans, the intake of water-soluble fibers and oligosaccharides (non-absorbable; data not shown) was higher than in omnivores and flexitarians (*p* ≤ 0.05). The intake of monosaccharides was similar in all the four diets. However, the intake of disaccharides was markedly lower in vegans than in flexitarians and vegetarians (*p* ≤ 0.05; data not shown).

In addition to the observed differences in the amount of dietary fat, its composition differs also markedly between the four study groups. The intake of saturated fatty acids (SFA) was high in omnivores and notably lower in both flexitarians and vegetarians (*p* ≤ 0.001). The lowest amounts of SFA were consumed in the vegan group. The omnivores consumed the highest amounts of monounsaturated fatty acids (MUFA) and vegan participants had the lowest intake (*p* ≤ 0.001). In contrast to SFA and MUFA, the intake of PUFA was low in omnivores, higher in vegetarians and highest in the vegan group (*p* ≤ 0.001; Table 2).

The intake of alpha linolenic acid (ALA) and linoleic acid (LA) were similar in all four groups and the consumption of palmitic acid, oleic acid, and docosapentaenoic acid (n-3) were higher in omnivores than in the vegetarian/vegan diets (*p* ≤ 0.05). The intake of stearic acid, arachidonic acid (n-6), eicosapentaenoic acid (n-3, EPA), and docosahexaenoic acid (n-3, DHA) differed also between the four groups, with decreasing of the intake in the following order: omnivores > flexitarians > vegetarians > vegans (*p* ≤ 0.01; Table 2).

The vegan group was characterized by the lowest daily intake of cholesterol which was ten times higher in omnivores (*p* ≤ 0.001). The average cholesterol intake in flexitarians, vegetarians and vegans matches the recommendations of the German Society of Nutrition (Table 2). The intakes of biotin, folic acid, vitamin B1, retinol equivalent, and vitamin C were similar between all studied diets and the average intakes complied with the recommendations of the German Society of Nutrition (Table 3), (17). The intake of niacin equivalent was the highest in omnivores (*p* ≤ 0.001). The average intake of pantothenic acid and vitamin A matches the recommendations only in omnivores. The lowest intake of vitamin A in the vegan group was partly compensated by a higher intake of beta carotene (*p* ≤ 0.001). Except for vitamin B2 and B12, the average intake of the B-vitamins complied with the recommendations for daily intake. The intake of vitamin B2 and B12 decrease as follows omnivores > flexitarians > vegetarians > vegans (*p* ≤ 0.05). In the vegan group, the average intake of vitamin B2 was below recommended levels. The recommended daily intake of Vitamin B12 (4 μg) was also not reached in vegetarians, flexitarians, and vegans (Table 3).

**Table 3:** Daily intake of vitamins (self-reports, 5 days)–NuEva-screening [Median/IQR; (Min–Max)].

		**Group 1**				**Group 2**				**Group 3**				**Group 4**			
	**Sex**	**Median**	**/**	**IQR**	**p**	**Median**	**/**	**IQR**	**p**	**Median**	**/**	**IQR**	**p**	**Median**	**/**	**IQR**	**p**
**Biotin** (μg)	All	58.3	/	30.7	a	53.0	/	27.1	a	51.6	/	25.7	a	47.8	/	22.1	a
30–60 μg/day^§^		(7.4–122.7)				(22.0–137.1)				(20.9–189.2)				(16.0–134.0)			
**Folic acid** (μg)	All	299	/	180	a	305	/	158	a	277	/	147	a	312	/	133	a
300 μg/day^§^		(52–833)		(101–671)		(103–1,209)		(144–1,606)									
**Niacin equivalent** (mg)	All	35.1	/	18.7	a	25.3	/	13.0	b	23.6	/	12.5	b,c	22.1	/	8.5	c
12–15 mg/day^§^		(5.7–85.2)				(11.3–56.1)				(7.0–97.6)				(6.7–51.6)			
**Pantothenic acid** (mg)	All	5.7	/	2.1	a	4.8	/	2.3	a,b	4.3	/	1.8	b	4.3	/	1.8	b
6 mg/day^§^		(0.82–13.50)				(1.77–24.0)				(1.55–15.97)				(1.95–12.94)			
**Vitamin A** (mg)	All	0.52	/	0.94	a	0.39	/	0.36	b	0.32	/	0.24	c	0.07	/	0.06	d
0.8–1.0 mg/day^§^		(0.11–3.48)				(0.02–4.07)				(0.08–0.78)				(0.01–0.57)			
**Beta carotene** (μg)	All	5,210	/	8,833	a	6,872	/	7,602	a,b	6,912	/	6,380	a	9,797	/	8,705	b
		(145–43,991)				(236–67,029)				(485–22,581)				(1,357–37,951)			
**Retinol equivalent** (μg)	All	1,614	/	2,379	a	1,592	/	1,418	a	1,513	/	1,079	a	1,742	/	1,522	a
800–1,000 μg/day^§^		(231–14,661)				(300–11,563)				(289–4,129)				(297–6,356)			
**Vitamin B**_**1**_ (mg)	m	1.94	/	0.89	a	1.60	/	0.70	a	1.48	/	0.91	a	1.54	/	0.94	a
1.0–1.2 mg/day^§^		(0.50–3.85)				(1.05–2.78)				(0.50–3.36)				(0.87–3.18)			
	w	1.34	/	0.46	a	1.27	/	0.46	a	0.93	/	0.67	a	1.26	/	0.68	a
		(0.50–3.12)				(0.17–2.37)				(0.22–4.74)				(0.46–2.84)			
	All	1.47	/	0.81	a	1.33	/	0.64	a,b	1.22	/	0.90	b	1.34	/	0.74	a,b
		(0.50–3.85)				(0.17–2.78)				(0.22–4.74)				(0.46–3.18)			
**Vitamin B**_**2**_ (mg)	m	1.84	/	0.70	a	1.79	/	0.76	a	1.44	/	0.53	a,b	1.01	/	0.35	b
1.1–1.4 mg/day^§^		(0.33–5.16)				(1.27–3.57)				(0.69–2.38)				(0.55–2.53)			
	w	1.66	/	0.56	a	1.36	/	0.63	b	1.20	/	0.50	b	0.79	/	0.32	c
		(0.56–5.28)				(0.58–3.18)				(0.44–3.09)				(0.33–2.22)			
	All	1.69	/	0.77	a	1.42	/	0.68	b	1.25	/	0.56	c	0.85	/	0.32	d
		(0.33–5.28)				(0.58–3.57)				(0.44–3.09)				(0.33–2.53)			
**Vitamin B**_**6**_ (mg)	All	2.04	/	0.96	a	1.79	/	0.96	b	1.47	/	0.64	b	1.77	/	0.87	b
1.1–1.4 mg/day^§^		(0.38–3.95)				(0.64–4.09)				(0.26–4.29)				(0.67–4.41)			
**Vitamin B**_**12**_ (μg)	m	6.43	/	4.38	a	3.39	/	2.29	b	2.05	/	2.06	c	0.39	/	1.09	d
4 μg/day^§^		(0.00–35.27)				(1.82–8.60)				(0.86–8.97)				(0–2.49)			
	w	5.36	/	3.74	a	2.81	/	1.92	b	1.77	/	1.33	c	0.37	/	0.57	d
		(1.61–42.63)				(0.36–10.83)				(0.26–9.81)				(0–3.75)			
	All	6.25	/	4.48	a	2.92	/	2.32	b	1.83	/	1.22	c	0.37	/	0.82	d
		(0.20–42.63)				(0.36–10.83)				(0.26–9.81)				(0–3.75)			
**Vitamin C** (mg)	All	131	/	79.8	a	147	/	103	a	124	/	94.6	a	161	/	83.9	a
95–110 mg/day^§^		(0–384)				(1–312)				(32–370)				(62–701)			
**Vitamin D** (μg)	All	2.28	/	1.83	a	1.71	/	1.74	b	1.67	/	1.57	b	0.94	/	1.27	c
20 μg/day^§^		(0.43–19.72)				(0.13–8.53)				(0.39–8.53)				(0.01–4.78)			
**Vitamin E** (mg)	All	9.16	/	4.80	a	11.1	/	5.79	a,b	13.3	/	8.51	b,c	14.5	/	8.00	c
12–14 mg/day^§^		(2.4–35.1)				(1.0–26.7)				(3.6–50.1)				(3.4–52.4)			
**Vitamin K** (μg)	All	154	/	167	a	198	/	180	a	195	/	181	a	243	/	184	b
60–70 μg/day^§^		(3–1,222)				(3–676)				(35–1,278)				(67–1,394)			

*Groups: 1 = omnivores, 2 = flexitarians, 3 = vegetarians, 4 = vegans*.

§ *Reference intake: (17). Significant influence of sex: vitamin B1, B2, B12*.

^*^Diet groups with different indices differ significantly (p < 0.05).

The dietary intake of vitamin D was far below the recommendations in all four groups, with the lowest daily intake in vegans. The average intake of vitamin E failed to reach the recommended values in both omnivores and flexitarians. The vitamin E intake in the vegetarian/vegan groups was higher than in omnivores (*p* ≤ 0.05). In all groups, vitamin K intake was 3 to 4 times higher than recommended, with the highest intake in vegans (*p* ≤ 0.01; Table 3).

The average intake of magnesium, potassium, and cooper were comparable between the four diets under consideration. In all study groups, the daily intake complied with recommended levels, except for potassium whose intake was subpar. The lower iron intake in flexitarians differ from the higher intake in vegans (*p* ≤ 0.05; Table 4). For most women, the recommended iron intake was not reached.

**Table 4:** Daily intake of minerals and trace elements (self-reports, 5 days)–NuEva-screening [Median/IQR; (Min–Max)].

		**Group 1**				**Group 2**				**Group 3**				**Group 4**			
	**Sex**	**Median**	**/**	**IQR**	**p**	**Median**	**/**	**IQR**	**p**	**Median**	**/**	**IQR**	**p**	**Median**	**/**	**IQR**	**p**
**Calcium** (mg)	All	870	/	520	a	884	/	367	a	862	/	357	a	576	/	266	b
1,000 mg/day^§^		(93–3,061)				(345–2,412)				(219–1,581)				(157–1,723)			
**Magnesium** (mg)	All	353	/	135	a	349	/	145	a	337	/	195	a	394	/	188	a
300–350 mg/day^§^		(121–812)				(174–689)				(68–1,085)				(137–894)			
**Sodium** (mg)	All	2,584	/	1,136	a	1,982	/	754	b	1,900	/	1,027	b	1,452	/	909	c
1,500 mg/day^§^		(896–6,322)				(693–5,593)				(409–4,286)				(198–3,181)			
**Potassium** (mg)	All	3,467	/	1,454	a	3,392	/	1,285	a	2,835	/	1,090	a	3,284	/	1,242	a
4,000 mg/day^§^		(683–6,923)				(1,171–7,198)				(660–7,313)				(1,316–6,972)			
**Chloride** (mg)	m	4,502	/	2,676	a	3,118	/	1,615	a	3,286	/	2,019	a	2,244	/	1,666	b
2,300 mg/day^§^		(1,470–8,358)				(2,257–6,021)				(1,561–6,878)				(906–3,868)			
	w	3,823	/	1,299	a	2,713	/	1,148	b	2,380	/	1,064	c	1,801	/	1,301	d
		(1,821–8,503)				(1,040–5,322)				(824–5,575)				(500–3,319)			
	All	3,926	/	1,764	a	2,731	/	1,157	b	2,418	/	1,304	b	1,898	/	1,349	c
		(1,470–8,503)				(1,040–6,021)				(824–6,878)				(500–3,868)			
**Phosphor** (mg)	All	1,476	/	461	a	1,271	/	586	b	1,156	/	520	b	940	/	372	c
700 mg/day^§^		(460–3,218)				(602–2,740)				(358–2,674)				(333–2,192)			
**Iron** (mg)	m	13.0	/	5.8	a	12.1	/	3.6	a	13.3	/	6.7	a	14.9	/	6.4	a
10–15 mg/day^§^		(4.5–28.8)				(7.7–17.0)				(6.8–27.7)				(7.5–27.8)			
	w	11.4	/	3.7	a	10.2	/	4.9	a	9.9	/	5.0	a	11.1	/	6.4	a
		(4.7–34.4)				(4.8–18.2)				(3.6–22.0)				(4.5–24.7)			
	All	12.1	/	4.2	a,b	10.9	/	4.6	a	10.6	/	6.2	a,b	12.6	/	6.5	b
		(4.5–34.4)				(4.8–18.2)				(3.6–27.7)				(4.5–27.8)			
**Copper** (μg)	m	1,956	/	909	a	1,919	/	796	a	2,361	/	1,502	a	2,161	/	865	a
1,000–1,500 μg/day^§^		(542–3,551)				(1,094–2,915)				(1,117–4,217)				(1,406–5,570)			
	w	1,654	/	630	a	1,649	/	653	a	1,615	/	912	a	1,804	/	633	a
		(741–3,725)				(773–2,927)				(376–5,145)				(627–3,422)			
	All	1,771	/	826	a	1,650	/	679	a	1740	/	1135	a	1,896	/	696	a
		(542–3,725)				(773–2,927)				(376–5145)				(627–5,570)			
**Manganese** (μg)	All	3,889	/	1,806	a	4,120	/	2,629	a	4553	/	3,642	a,b	5,511	/	3,421	b
2,000–5,000 μg/day^§^		(1,887–10,538)				(1,581–16,342)				(1,375–18,376)				(1,923–13,948)			
**Zinc** (mg)	m	12.6	/	9.6	a	11.1	/	2.7	b	9.9	/	5.9	b	9.3	/	3.3.	b
11–16 mg/day^§^		(3.7–25.0)				(7.0–13.9)				(4.6–22.2)				(4.0–15.4)			
	w	11.2	/	4.7	a	9.2	/	4.0	b	7.9	/	3.5	b	6.7	/	2.5	c
		(4.0–24.8)				(4.2–18.0)				(2.8–20.2)				(2.6–17.7)			
	All	12.0	/	5.9	a	9.7	/	4.2	b	8.1	/	3.6	b	7.1	/	3.3	c
		(3.7–25.0)				(4.2–18.3)				(2.8–22.2)				(2.6–17.7)			

*Groups: 1 = omnivores, 2 = flexitarians, 3 = vegetarians, 4 = vegans. Adjusted for BMI: Jodine (μg)*.

§ *Reference intake: (17). Significant influence of sex: chloride, iron, copper, zinc*.

The selenium intake was not calculated because the nutritional software (PRODI^®^) does not provide any information on the selenium levels in foods. The iodine intake was not calculated because the additional intake by fortified table salt was unknown. ^*^Diet groups with different indices differ significantly (p < 0.05).

In group 1 to 3, the average calcium intake was almost at recommended level but was lower in vegans (576/266 mg; *p* ≤ 0.05). In women, the intake of chloride varied in the following order: omnivores > flexitarians/vegetarians > vegans (*p* ≤ 0.05). The comparably high intake of phosphor in omnivores, but also in flexitarians and in vegetarians differed from the lower intake in the vegan group (*p* ≤ 0.01).

In groups 2 to 4 the average intake of zinc was lower than recommended, whereby the higher zinc intake in omnivores differed significantly from the lower intakes in the groups 2 to 4 (*p* ≤ 0.001). The lowest zinc intake in the vegan subjects varied from the higher intake in omnivores, flexitarians, and vegetarians, respectively (*p* ≤ 0.01). In total, the average intakes of the vitamins B1, B2, B12, chloride, iron, copper, and zinc were higher in men than in women (*p* ≤ 0.01; Tables 3, 4).

### Anthropometric Data

The highest body weight in omnivores differed significantly from the lower ones in groups 2 to 4 (*p* ≤ 0.05; Table 5). BMI depended on age. After adjustment for age, the higher BMI values in omnivores differed from the lower ones in groups 2 to 4 (*p* ≤ 0.05). Comparable differences were detected for waist circumference (*p* ≤ 0.05; Table 5).

**Table 5:** Anthropometric data, body composition and blood lipids – NuEva-screening [Median/IQR; (Min–Max)].

		**Group 1**				**Group 2**				**Group 3**				**Group 4**			
**Parameter**	**Sex**	**Median**	**/**	**IQR**	**p**	**Median**	**/**	**IQR**	**p**	**Median**	**/**	**IQR**	**p**	**Median**	**/**	**IQR**	**p**
Anthropometric data																	
**Body weight**	m	80.7	/	14.6	a	68.4	/	7.5	b	79.2	/	16.1	a,b	73.2	/	8.6	a,b
(kg)		(68.0–124.6)				(62.6–90.8)				(56.5–94.6)				(50.3–92.9)			
	w	71.8	/	17.8	a	62.6	/	8.6	b	62.6	/	13.6	b,c	57.9	/	10.0	c
		(46.8–100.9)				(51.1–91.4)				(47.5–92.5)				(47.6–80.0)			
	All	73.7	/	15.7	a	64.2	/	9.3	b	65.4	/	14.4	b	59.4	/	17.0	b
		(46.8–124.6)				(51.1–91.4)				(47.5–94.6)				(47.6–92.9)			
**BMI**	m	24.6	/	4.0	a	22.5	/	2.1	b	23.6	/	3.9	a,b	22.6	/	2.4	b
(kg/m^2^)		(19.8–40.5)				(18.8–24.9)				(18.8–28.6)				(17.0–26.9)			
	w	25.4	/	5.7	a	22.1	/	3.5	b	21.6	/	3.3	b,c	20.8	/	3.1	c,d
		(18.5–33.7)				(18.3–32.2)				(17.3–29.9)				(17.1–27.3)			
	All	24.6	/	4.9	a	22.1	/	3.4	b	22.4	/	3.5	b	21.6	/	3.4	b
		(18.5–40.5)				(18.3–32.2)				(17.3–29.9)				(17.0–27.3)			
**Waist**	All	80.0	/	14.0	a	73.0	/	11.8	b	74.0	/	11.0	b	72.5	/	11.5	b
**circumferences** (cm)		(53–165)				(40–123)				(51–116)				(52–99)			
Body composition																	
**Body cell mass**	m	36.6	/	5.4	a	33.1	/	6.0	a	33.3	/	4.2	a	34.6	/	6.1	a
(BCM, kg)		(30.1–52.1)				(28.7–40.6)				(29.0–39.5)				(24.2–43.3)			
	w	25.9	/	4.9	a	23.9	/	3.9	b	24.5	/	2.7	b	23.5	/	4.6	b
		(20.0–33.8)				(18.8–32.3)				(19.4–29.9)				(18.0–28.7)			
	All	28.9	/	10.4	a	25.0	/	6.8	b	25.5	/	6.3	b	24.5	/	6.3	b
		(20.0–52.1)				(18.8–40.6)				(19.4–39.5)				(18.0–43.3)			
**Extracellular mass**	m	25.8	/	6.3	a	22.8	/	4.3	a	28.4	/	6.8	a	25.0	/	6.7	a
(ECM, kg)		(18.6–35.1)				(18.6–36.3)				(18.4–34.4)				(19.6–33.0)			
	w	21.9	/	4.3	a	21.1	/	3.0	a	20.3	/	3.7	a	20.2	/	3.2	a
		(13.2–30.8)				(14.9–27.0)				(14.6–30.1)				(14.0–27.4)			
	All	22.9	/	5.6	a	21.6	/	3.4	a	21.6	/	6.1	a	20.9	/	5.1	a
		(13.2–35.1)				(14.9–36.3)				(14.6–34.4)				(14.0–33.0)			
**ECM/BCM**	m	0.72	/	0.15	a	0.71	/	0.11	a,b	0.85	/	0.20	c	0.78	/	0.19	a,c
		(0.5–1.1)				(0.5–1.1)				(0.6–1.1)				(0.5–1.0)			
	w	0.86	/	0.23	a	0.88	/	0.16	a	0.86	/	0.23	a	0.93	/	0.18	a
		(0.5–1.1)				(0.5–1.2)				(0.6–1.3)				(0.5–1.2)			
	All	0.80	/	0.30	a	0.85	/	0.24	a	0.86	/	0.23	a	0.86	/	0.25	a
		(0.5–1.1)				(0.5–1.2)				(0.6–1.3)				(0.5–1.2)			
**Metabolic rate**	m	1,770	/	170	a	1,665	/	190	a	1,670	/	128	a	1,710	/	190	a
(kcal)		(1,570–2,260)				(1,520–1,900)				(1,530–1,860)				(1,380–1,980)			
	w	1,440	/	150	a	1,370	/	123	b	1,390	/	88	b	1,355	/	143	b
		(1,250–1,680)				(1,210–1,640)				(1,230–1,560)				(1,190–1,520)			
	All	1,530	/	333	a	1,405	/	215	b	1,425	/	205	b	1,390	/	195	b
		(1,250–2,260)				(1,210–1,900)				(1,230–1,860)				(1,190–1,980)			
**Body fat** (kg)	m	18.1	/	8.0	a	12.4	/	4.3	b	14.1	/	6.1	a,b	13.0	/	5.7	b
		(8.7–39.9)				(8.6–19.7)				(7.0–31.3)				(4.7–18.9)			
	w	23.1	/	13.4	a	17.5	/	8.2	b	16.5	/	8.5	b	14.5	/	6.5	b
		(8.8–44.0)				(8.4–38.7)				(7.7–38.3)				(8.3–31.6)			
	All	20.4	/	12.6	a	16.6	/	7.6	b	16.1	/	9.0	b	14.4	/	6.6	b
		(8.7–44.0)				(8.4–38.7)				(7.0–38.3)				(4.7–31.6)			
**Body water** (l)	m	46.5	/	7.8	a	41.1	/	6.2	a	44.0	/	6.5	a	43.7	/	6.9	a
		(37.4–62.0)				(35.5–53.1)				(36.2–53.6)				(33.5–55.4)			
	w	35.2	/	4.5	a	32.4	/	4.0	b	32.8	/	4.1	b	31.4	/	3.6	b
		(27.3–44.3)				(27.6–41.7)				(26.8–39.7)				(26.6–39.0)			
	All	37.5	/	9.5	a	33.8	/	7.4	b	34.2	/	9.9	b	32.7	/	7.0	b
		(27.3–62.0)				(27.6–53.1)				(26.8–53.6)				(26.6–55.4)			
**Lean body mass**	m	63.6	/	10.6	a	56.0	/	8.5	a	60.2	/	8.7	a	59.7	/	9.5	a
(LBM, kg)		(51.0–84.7)				(48.5–72.5)				(49.5–73.3)				(45.8–75.6)			
	w	48.0	/	6.1	a	44.2	/	5.4	b	44.7	/	5.7	b	42.9	/	4.9	b
		(37.3–60.5)				(37.6–56.9)				(36.6–54.2)				(36.4–53.2)			
	All	51.1	/	12.9	a	46.1	/	10.1	b	46.7	/	13.6	b	44.7	/	9.5	b
		(37.3–84.7)				(37.6–72.5)				(36.6–73.3)				(36.4–75.6)			
**Phase angle**	m	7.50	/	1.50	a	7.60	/	0.95	a,b	6.45	/	1.25	c	7.00	/	1.73	a,c
(°)		(5.3–10.5)				(5.4–9.9)				(5.2–9.0)				(5.7–9.9)			
	w	6.40	/	1.50	a	6.25	/	1.03	a	6.45	/	1.55	a	6.00	/	1.03	a
		(5.1–9.8)				(4.9–10.0)				(4.6–8.8)				(4.7–9.5)			
	All	6.85	/	2.15	a	6.40	/	1.70	a	6.45	/	1.50	a	6.40	/	1.63	a
		(5.1–10.5)				(4.9–10.0)				(4.6–9.0)				(4.7–9.9)			
**Cell amount**	m	58.3	/	5.4	a	58.7	/	3.9	a,b	54.2	/	5.6	c	56.2	/	6.6	a,c
(amount BCM in LBM		(48.3–68.1)				(48.9–66.4)				(47.9–63.8)				(50.6–66.5)			
%)	w	54.0	/	6.6	a	53.3	/	4.7	a	54.1	/	6.8	a	52.0	/	4.7	a
		(47.5–66.1)				(45.8–66.8)				(44.5–63.1)				(44.7–65.4)			
	All	55.7	/	9.1	a	54.0	/	7.2	a	54.1	/	6.8	a	53.8	/	7.1	a
		(47.5–68.1)				(45.8–66.8)				(44.5–63.8)				(44.7–66.5)			
Blood lipids																	
**Total cholesterol**	All	4.90	/	1.18	a	4.63	/	1.14	a,b	4.54	/	1.02	b	3.71	/	0.77	c
(mmol/l)		(3.5–7.6)				(3.0–8.6)				(2.9–7.1)				(2.6–5.5)			
**HDL cholesterol** (mmol/l)	m	1.27	/	0.36	a	1.38	/	0.21	a	1.28	/	0.32	a	1.32	/	0.36	a
		(0.8–2.0)				(0.9–2.0)				(1.0–2.0)				(0.9–1.9)			
	w	1.59	/	0.52	a	1.62	/	0.52	a	1.65	/	0.45	a	1.53	/	0.47	a
		(1.0–2.6)				(1.0–2.3)				(1.0–2.8)				(0.8–2.3)			
	All	1.47	/	0.53	a	1.59	/	0.49	a	1.57	/	0.56	a	1.47	/	0.47	a
		(0.8–2.6)				(0.9–2.3)				(1.0–2.8)				(0.8–2.3)			
**Total cholesterol /**	All	3.16	/	1.39	a	2.91	/	1.00	a,b	2.80	/	1.22	b	2.58	/	0.64	c
**HDL cholesterol**		(1.9–8.5)				(1.9–6.4)				(1.9–5.1)				(1.6–4.4)			
**LDL cholesterol**	All	2.83	/	0.93	a	2.62	/	1.05	a,b	2.66	/	1.17	b	2.06	/	0.72	c
(mmol/l)		(1.8–5.3)				(1.2–6.4)				(1.4–4.4)				(0.7–3.4)			
**LDL cholesterol /**	All	1.86	/	1.22	a	1.74	/	0.92	a,b	1.53	/	1.13	b,c	1.40	/	0.69	c
**HDL cholesterol**		(0.7–5.0)				(0.8–4.7)				(0.8–3.5)				(0.4–2.9)			
**Triacylglycerols**	All	0.82	/	0.72	a	0.87	/	0.51	a	0.82	/	0.36	a	0.71	/	0.35	a
(mmol/l)		(0.4–3.3)				(0.4–3.0)				(0.4–2.8)				(0.3–2.4)			
**Malondialdehyde- modified LDL**	All	48.7	/	25.5	a	43.2	/	34.9	a,b	48.9	/	28.6	a	37.6	/	19.0	b
(U/l)		(13.5–132)				(4.28–128)				(15.9–103)				(13.9–96.1)			
**Apolipoprotein A1**	All	1.48	/	0.37	a	1.51	/	0.36	a	1.50	/	0.30	a	1.43	/	0.36	b
(g/l)		(1.0–2.5)				(0.9–2.3)				(1.0–2.7)				(1.0–2.0)			
**Apolipoprotein B**	All	0.85	/	0.32	a	0.83	/	0.34	a,b	0.80	/	0.35	b	0.64	/	0.19	c
(g/l)		(0.5–1.8)				(0.5–2.1)				(0.5–1.4)				(0.4–1.1)			
**Apolipoprotein B /**	m	0.61	/	0.29	a	0.63	/	0.43	a	0.64	/	0.32	a	0.51	/	0.17	a
**Apolipoprotein A1**		(0.4–1.3)				(0.4–1.1)				(0.3–1.0)				(0.3–0.8)			
	w	0.53	/	0.24	a	0.52	/	0.22	a	0.46	/	0.18	a,b	0.43	/	0.17	b
		(0.3–1.1)				(0.3–1.3)				(0.3–1.0)				(0.2–0.7)			
	All	0.59	/	0.26	a	0.54	/	0.25	a,b	0.49	/	0.26	b,c	0.45	/	0.17	c
		(0.3–1.3)				(0.3–1.3)				(0.3–1.0)				(0.2–0.8)			

*Adjusted for age: BMI, total cholesterol, LDL cholesterol, apolipoprotein A1, apolipoprotein B. Adjusted for BMI: waist circumferences. Significant influence of sex: weight, BMI, body cell mass, extracellular mass, BCM/ECM, metabolic rate, body fat, body water, lean body mass, phase angle, cell amount, HDL cholesterol, apolipoprotein A1/ apolipoprotein B*.

^*^Diet groups with different indices differ significantly (p < 0.05).

The body composition differed significantly between men and women (Table 5). Extracellular mass (ECM) includes all the metabolically inactive body components, whereas the body cell mass (BCM) describes the metabolically active tissues of the body. Thus, the ECM/BCM ratio is a highly sensitive index of malnutrition (18). In men, the BCM did not differ significantly between the four studied groups. In women and the entire collective, the higher values in omnivores varied from the lower ones in group 2 to 4 (*p* ≤ 0.05). The ECM and the ECM/BCM ratio did not differ between the four diet groups, except the slightly lower ratios in omnivorous and flexitarian men in comparison to the higher ratios in vegetarian men (*p* ≤ 0.05; Table 5). In omnivores, the basal metabolic rate was on average 100 kcal higher than in flexitarians, vegetarians, and vegans (*p* ≤ 0.05). The body fat mass was higher in omnivorous men in comparison to flexitarian and vegan men. In women and the entire study population, the higher values in omnivores differed from the values measured in the groups 2 to 4 (*p* ≤ 0.05).

In men of the studied diets, the body water fraction was similar. The highest amounts of approx. 35 L in omnivorous women differed from the values measured in the other three groups which varied between 28–42 L (*p* ≤ 0.05; Table 5). Comparable differences between the diets studied were also identified for the lean body mass (LBM) which highly correlates with muscle mass. LBM is defined as the difference between total body weight and body fat. The highest LBM was measured in omnivores with approx. 53 kg which differed from the lower LBM in groups 2 to 4 (*p* ≤ 0.05; Table 5).

The phase angle (PhA; °) normally ranges between 5 to 7°. Values below the reference limit were found in groups 2 to 4 (Table 5). The lowest PhA in vegetarian men differed significantly from the higher PhA measured in omnivorous and flexitarian men (*p* ≤ 0.05). Similar differences were found for the cell amount which describes the amount of BCM in the LBM (Table 5). The highest cell amount in omnivorous and flexitarian men differed from the lower levels in vegetarian men (*p* ≤ 0.05; Table 5). In women and the entire study population cell amount and PhA were comparable between the four groups investigated (Table 5).

### Blood Lipids and Vitamin B12 Status

The highest concentrations on total cholesterol, total cholesterol/HDL cholesterol ratio, LDL cholesterol, LDL cholesterol/HDL cholesterol ratio, apolipoprotein B, and apolipoprotein B/apolipoprotein A1 ratio in omnivores differ from the lower values in vegetarians and vegans (*p* ≤ 0.05). Apolipoprotein A1 concentration in groups 1 to 3 were higher than in vegans (*p* ≤ 0.05). HDL cholesterol and triacylglycerols did not differ between the four groups (Table 6). The higher concentrations of malondialdehyde-modified LDL in omnivores and vegetarians differ from the lower ones in vegans (*p* ≤ 0.05; Table 6).

**Table 6:** Vitamins, minerals and trace elements in plasma/serum and 24h urine – NuEva-screening (Median / IQR; (Min - Max)).

		**Group 1**				**Group 2**				**Group 3**				**Group 4**			
**Parameter**	**Sex**	**Median**	**/**	**IQR**	***p***	**Median**	**/**	**IQR**	***p***	**Median**	**/**	**IQR**	***p***	**Median**	**/**	**IQR**	***p***
Plasma / serum																	
**Biotin**	All	249	/	108	a	305	/	161	b	284	/	136	a,b	291	/	166	b
(ng/l)		(94–1,000)				(143–1,000)				(62–1,000)				(101–1,000)			
**Folate**	All	7.20	/	6.00	a,b	8.65	/	4.18	a,b	8.10	/	3.90	a	10.40	/	5.03	b
(μg/l)		(2.2–16.9)				(3.2–16.5)		(2.9–16.9)		(3.7–18.3)							
**Vitamin B**_**12**_	All	242	/	94	a	246	/	119	a	208	/	110	b	213	/	161	a,b
(pmol/l)		(109–567)				(116–508)				(110–966)				(128–712)			
**Holo-Transcobalamine**	All	80.8	/	44.1	a	73.9	/	35.1	a	54.9	/	29.8	b	54.9	/	47.6	c
(pmol/l)		(39–227)				(26–180)				(11–356)				(14–327)			
**Homocysteine**	All	9.5	/	4.4	a	10.5	/	4.1	a	10.2	/	4.4	a	10.0	/	3.7	a
(μmol/l)		(4.4–21.2)				(5.3–19.2)				(5.2–33.5)				(3.7–37.8)			
**Methyl malonic acid**	All	17.0	/	8.5	a	20.0	/	10.0	a	21.0	/	13.0	a	18.5	/	12.3	a
(μg/l)		(9–65)				(8–57)				(9–82)				(7–64)			
**4cB12 score** ^§^	All	0.34	/	0.58	a	0.24	/	0.52	a,c	0.02	/	0.75	c	0.08	/	0.89	b,c
		(−0.51 to 1.33)				(−0.66 to 1.45)				(−2.05 to 2.07)				(−1.44 to 1.52)			
**Vitamin B**_**1**_	All	137.2	/	34.2	a,b	140.0	/	37.6	a	130.3	/	37.6	b	133.0	/	33.3	a,b
(nmol/l)		(79 – 235)				(72–215)				(63–275)				(91–208)			
**Vitamin B**_**2**_	All	230	/	54.3	a	247	/	37.0	b	225	/	56.0	a,c	220	/	44.5	a,c
(μg/l)		(150–334)				(175–343)				(155–335)				(147–318)			
**Vitamin B**_**6**_	All	51.7	/	40.8	a	54.6	/	28.6	a	48.7	/	29.1	a	54.8	/	30.8	a
(nmol/l)		(20–264)				(18–187)				(14–257)				(15–194)			
**Vitamin C**	All	6.9	/	3.7	a	7.8	/	5.8	a,b	8.8	/	4.7	b	10.4	/	4.1	c
(mg/l)		(0.4–13.1)				(1.6–19.5)				(0.6–16.6)				(3.0–20.4)			
**Vitamin A**	All	1.61	/	0.62	a	1.75	/	0.58	a	1.67	/	0.59	a	1.35	/	0.42	b
(μmol/l)		(0.9–3.1)				(1.0–3.0)				(1.0–2.9)				(0.9–2.9)			
**Vitamin D**	All	70.7	/	21.6	a	65.4	/	26.6	a	68.3	/	34.3	a	65.0	/	22.3	a
(nmol/l)		(17–134)				(34–118)				(18–145)				(16–181)			
**Vitamin E**	All	26.7	/	8.9	a	27.1	/	7.8	a	25.0	/	7.3	a,b	24.0	/	6.8	b
(μmol/l)		(17–72)				(17–60)				(14–44)				(13–47)			
**Ferritin**	All	80.1	/	89.6	a	31.3	/	44.2	b	31.2	/	19.6	b	29.9	/	39.8	b
(μg/l)		(3.1–455)				(2.5–223)				(4.5–267)				(1.5–169)			
**Transferrin**	All	2.5	/	0.5	a	2.8	/	0.78	b	2.8	/	0.5	b	2.8	/	0.5	b
(g/l)		(2.0–3.9)				(1.9–4.7)				(2.0–3.9)				(1.8–4.1)			
**Transferrin saturation**	All	28.5	/	13.2	a	26.2	/	18.6	a	27.0	/	13.3	a	30.9	/	20.1	a
(%)		(6.4–88.0)				(2.9–57.7)				(6.6–60.0)				(7.8–73.0)			
**24h urine**																	
**Magnesium**	All	4.30	/	2.10	a	4.40	/	1.93	a	4.80	/	1.60	a	4.90	/	2.20	a
(mmol/24h)		(1.0–10.6)				(1.4–9.5)				(1.0–8.7)				(1.3–9.9)			
**Sodium**	All	143	/	79	a	113	/	71	a	146	/	80	a	128	/	88	a
(mmol/24h)		(61–291)				(40–299)				(48–282)				(42–346)			
**Selenium**	All	0.25	/	0.19	a	0.19	/	0.13	b	0.20	/	0.09	b	0.16	/	0.12	b
(μmol/ 24h)		(0.07–0.77)				(0.06–0.76)				(0.07–0.66)				(0.06–0.91)			
**Zinc**	m	10.75	/	3.33	a	8.30	/	8.00	a	8.25	/	4.53	a	6.05	/	3.55	a
(μmol/24h)		(3.6–32.8)				(3.4–19.7)				(2.8–13.6)				(4.3–13.4)			
	w	5.85	/	4.23	a	5.20	/	3.08	a	5.60	/	4.20	a	4.20	/	2.70	b
		(3.2–27.2)				(1.8–14.6)				(1.7–18)				(0.8–9.5)			
	All	7.85	/	5.58	a	5.50	/	4.60	b,c	6.10	/	3.90	b	5.00	/	3.30	c
		(3.2–32.8)				(1.8–19.7)				(1.7–18)				(0.8–13.4)			
**Iodine**	All	53.0	/	47.5	a	52.0	/	35.5	a,b	42.0	/	27.0	a,b	21.5	/	16.8	b
(μg/l)		(17–268)				(13–192)				(6–335)				(8–509)			

*Significant influence of sex: zinc*.

*Adjusted for age: vitamin E*.

^*^Diet groups with different indices differ significantly (p <0.05).

§ *4cB12 score–combined index of B12 deficiency (normal range:−0.5 - 1.0)*.

Vitamin B12 concentrations in plasma were higher in omnivores and flexitarians compared to vegetarians (Table 6). Vitamin B12 concentrations below the reference range were found in all groups studied. Holotranscobalamin (holoTC) varied between 11 to 356 pmol/l with the lowest concentrations in vegetarians and vegans and the highest means in both omnivores and flexitarians (*p* ≤ 0.05; Table 6). Concentrations below the reference range of 37.5 pmol/l were found in individuals from all studied groups, whereby the lowest concentrations observed in groups 1 and 2 ranged between 26 to 39 pmol/l and in the vegetarian/vegan groups lowest concentration between 11 to 14 pmol/l were detected.

Plasma methylmalonic acid and homocysteine concentrations were comparable between the studied groups (Table 6). The 4cB12 score was calculated from the above-mentioned parameters as a combined indicator of vitamin B12 status (19). Altogether, the lowest 4cB12 score in the vegetarian group (0.02/0.75) differed from the higher index calculated in omnivores (0.34/0.58) and flexitarians (0.24/0.52; *p* ≤ 0.05; Table 6). The lower score in vegans (0.08/0.89) varied also from the score in omnivores (*p* ≤ 0.05; Table 6).

### Concentrations of Further Vitamins, Minerals, and Trace Elements in Plasma or Serum

In omnivores concentrations of vitamin A and vitamin E were higher than in vegans (*p* ≤ 0.05 Table 6). The lowest vitamin A concentration observed in the vegan group differed from the higher means in the groups 1 to 3 (*p* ≤ 0.05; Table 6). Vitamin E concentrations were adjusted for age and were on average 29 μmol/l in both omnivores and flexitarians, respectively and thus higher compared to the vegan group (*p* ≤ 0.01; Table 6).

The lowest vitamin B1 concentrations were detected in the vegetarian group which differ from the higher concentrations in flexitarians (*p* ≤ 0.05; Table 6). Vitamin B2 concentrations ranged from 147 to 343 μg/l whereby the higher concentrations detected in flexitarians differ from the lower concentrations in omnivores and vegetarian/vegan diets (*p* ≤ 0.001; Table 6).

The folate concentrations varied between 2.2 to 18.3 μg/l with the highest values recorded in the vegan group (on average 10 μg/l; Table 6). The folate concentrations in vegetarians were lower than in vegans (*p* ≤ 0.05). Biotin values in omnivores were lower compared to flexitarians and vegans, respectively (Table 6). In comparison to omnivores the detected vitamin C concentrations were higher in vegetarian/vegan diets (*p* ≤ 0.05; Table 6).

Vitamin B6 and vitamin D concentrations ranged between 14 to 264 nmol/l and 16 to 181 nmol/l, respectively, without significant differences between the studied groups.

In the NuEva study population, concentrations of calcium, potassium, iron, and the iron saturation were comparable between the four diets studied.

Highest ferritin values were observed in the omnivores compared to flexitarians, vegetarians, and vegans (*p* ≤ 0.01; Table 6). The lowest ferritin concentration of 1.5 μg/l was detected in a vegan woman, whereas low levels between 2.5 to 4.5 μg/l were also detected in women of group 1, 2 and 3. Ferritin levels above the upper limit of normal were observed in both men and women of the omnivorous group only. The lowest transferrin concentrations were measured in omnivores compared to groups 2, 3 and 4 (*p* ≤ 0.01; Table 6).

### Concentrations of Albumin, Creatinine, Minerals, and Trace Elements in 24h Urine Collection

Albumin and creatinine concentrations in 24h urine ranged from 5–82 mg/l and 1.4–21.9 mmol/l, respectively. The lowest values observed in vegetarians and vegans, diffed from omnivorous group's concentrations, respectively (*p* ≤ 0.05; data not shown).

Magnesium and sodium in 24h urine was comparable between the groups studied. With regard to trace elements, highest zinc, and selenium concentrations were identified in omnivores differing from the lower ones in flexitarians, vegetarians, and vegans (*p* ≤ 0.05; Table 6). The highest urinary iodine concentrations in the omnivores differ from the lower values in vegans (*p* ≤ 0.05; Table 6).

## Discussion

### Nutrient Intake and Cardiovascular Risk Factors

The data on nutrient intake showed substantial differences between meat-eaters and the vegetarian/vegan diets, with the strongest differences between omnivores and vegans. In line with data from Clarys et al. (20) the comparable high energy intake in omnivores differs from the lower energy content of the diet in vegans. In contrast, Weikert et al. (21) found no differences in energy intake between German vegans and omnivores. The higher energy intake effects body weight as in comparison to flexitarians, vegetarians, and vegans, omnivores showed a higher body weight (approx. 10 kg), associated with a 3 to 4 points increase of the BMI index and a 10 to 13 cm wider waist circumference. Matsumoto et al. (22) described also lower values of BMI and waist circumference in the vegetarian subgroup of the non-Hispanic whites in the Adventist Health Study-2. Overall, in the NuEva screening differences for body weight, BMI, LBM, BCM, body fat, and basal metabolic rate were detected in the following order: omnivores > flexitarians > vegetarians > vegans (omnivores vs. vegans: ≤ 0.05). We assume that the differences of anthropometric parameters and body composition relates to the characteristic intake of energy, fat, and protein which was calculated based on the dietary protocols. We would neglect the influence of physical activity as strenuous physical activity (> 15 h per week) was an exclusion criterion (13). The data available from the activity protocol indicate for a low to moderate physical activity of the NuEva participants (data not shown).

Our findings on weight and body composition are in accordance with previous data and highlighted the potential of plant-oriented dietary diets for weight management and therefore prevention of CVD (23, 24).

In line with previous findings, the intake of energy, major nutrients, dietary fibers and SFA differs strongly between the four groups with the most significant dissimilarity between omnivores and vegans (20, 21, 25). These characteristic differences may have an impact on the development of risk factors for non-communicable diseases, particularly CVD (26–29). In this context, the reduction of energy and SFA intake plays a key role in prevention of CVD. Thus, consuming less than 10% or in case of hypercholesterolemia, even less than 7% of total calorie intake (en%) from SFA is recommended by both European and American experts (17, 30). In the NuEva study population, only the vegans met these recommendations.

The NuEva study was able to show the sharp contrast between the intake of energy, dietary fibers, fat and SFA between omnivores and vegans which is accompanied by significantly higher concentrations of blood lipids in omnivores. Malondialdehyde-modified LDL is a marker for oxidative stress which is associated with atherosclerotic cardiovascular diseases (31). The lowest concentrations detected in vegans may related to a higher intake of antioxidative compounds, such as carotenoids, vitamin C, vitamin K, and vitamin E in this group.

In summary, our data indicate for the highest cardioprotective potential of the vegan diet.

### Critical Nutrients in Omnivores, Flexitarians, Vegetarians, and Vegans

In omnivores, the average intake of energy, total fat, SFA, cholesterol, disaccharides, total sugar, and purines was higher than recommended by the German Society of Nutrition (17) and the intake of carbohydrates, particularly dietary fiber, PUFA mainly n-3 PUFA, potassium, vitamin D, vitamin E was lower than recommended.

For flexitarians, the following critical nutrients were identified with intakes higher than recommended: total fat, SFA, disaccharides, total sugar. In this group, the average intake of carbohydrates, particularly dietary fiber, PUFA particularly n-3 PUFA, pantothenic acid, vitamin B12, vitamin D, vitamin E, iron (woman), potassium, and zinc fell below the DGE recommendations (17).

In vegetarians, the intake of total fat, SFA, disaccharides, and total sugar was also above the recommendations while the intake of total protein, PUFA particularly n-3 PUFA, pantothenic acid, vitamin B12, vitamin D, calcium, iron (women), potassium, and zinc were below the recommendations for adequate nutrient intake (17).

In vegans, the mean intake of total sugar was also higher than recommended. In this diet form, the sugar intake mainly arose from fruits while consumption of sugar such as chocolate or gummy bears was comparably lower, as these foods often contain animal-based ingredients (20, 32). Still, the average intake of total protein, PUFA, particularly n-3 PUFA, pantothenic acid, vitamin B2, vitamin B12, vitamin A, vitamin D, calcium, potassium, iron (women), and zinc was markedly lower than recommended by the DGE (17). The intake of calcium was especially low in the vegan diet, as no dairy products are consumed. Clarys et al. (20) reported a mean vegans' calcium intake of 738 ± 456 mg/day and Weikert et al. (21) described a mean intake of 899 mg/day. The lower intakes in the present study are in accordance with data available from the EPIC-Oxford vegans (men: 603 ± 232 mg/day; women: 586 ± 226 mg/day), (33). In the EPIC oxford cohort, the percentage of subjects consuming less than 700 mg/day calcium was 15.0 for meat eaters, 15.9 for fish eaters, 18.6 for vegetarians and 76.1 for vegans which is similar to the NuEva screening. Appleby et al. (33) described a 30% higher fracture rate in vegans which disappeared when the analysis was conducted with all participants who consume less than 525 mg calcium/day.

The average intake of iron was below the recommendations in flexitarians and vegetarians but almost reached the optimal levels in both omnivores and vegans. Considering the lower bioavailability of non-heme iron (iron from plant origin), the iron intake in vegetarian/vegan diets should be 1.8 times higher than in omnivores diets (34). In accordance with the data from Kristensen et al. (25), vegan men nearly reached these recommended amounts.

In the diets under consideration, the average concentrations of all vitamins, minerals and trace elements analyzed, except for vitamin D and iodine, were within the reference range. Previous data indicate undersupply for vitamin D as a general problem independently from the diets studied (35, 36).

Evident from the dietary records, the intake of vitamin B2 and B12 decreased significantly in the following order omnivores > Flexitarians > vegetarians > vegans, because dairy products, fish and meat are the main food sources of these vitamins. Schüpbach et al. (37) found also higher average vitamin B2 levels in omnivores (92.0 ± 44.8 nmol/l; *n* = 100), whereby the difference to the lower values in vegetarians (82.4 ± 42.4 nmol/l; *n* = 53) and vegans (79.8 ± 41.7 nmol/l; *n* = 53) was not significant.

Undersupply with vitamin of B12 is a well-known problem in vegetarian/vegan diets as only animal-based foods deliver relevant amounts of active vitamin B12 (38). A 4cB12 score between −1.5 and −0.5 indicates for a low vitamin B12 supply and was calculated in one omnivorous participant, two flexitarians, ten vegetarians and ten vegans. A 4cB12 index between −1.5 and −2.5 indicates a potential B12 deficiency and was found in one vegetarian woman. In contrast to the findings from Weikert et al. (21) which described comparable 4cB12 indices between vegans (0.54) and omnivores (0.42; *p* = 0.62), the low 4cB12 indices in vegetarians and vegans differed significantly from the index calculated in omnivores. Thus, our data indicate for the risk of undersupply with vitamin B12 in the vegetarian/vegan groups which can manifests in macrocytic anemia or neurological impairments and can lead to irreversible neurological damage if undetected (39, 40).

Depletion of iron stores, defined by ferritin concentrations below 20/30 μg/l for women and men, were detected in 20 and 0% of the omnivorous group, 43/14% flexitarians, 28/17% vegetarians, and 42/6% vegans, respectively. Since, ferritin is also an acute-phase reactant, its synthesis is upregulated by infection or inflammation (41). In the NuEva collective, c-reactive protein (CRP) concentrations between 3,5 mg/l and 22 mg/l were observed in six participants of the omnivorous group, four flexitarians, seven vegetarians and two vegans (data not shown). In this subgroup, hemoglobin, MCV and transferrin saturation were within the normal range (*n* = 15), except for two participants showing hemoglobin values below 7.6 mmol/l, one omnivorous participant and one vegan with an additional transferrin saturation below 16%. Moreover, in one vegetarian and one vegan only transferrin saturation was below 16%, indicating latent iron deficiency.

In accordance with the literature, men were marked by two to threefold higher ferritin concentrations than women (42). In addition, the NuEva screening shows significantly higher ferritin concentrations and lower transferrin concentrations in omnivores in comparison to the other studied diets. The obvious difference in ferritin levels between omnivores and vegetarian/vegan diets were also described by Schüpbach et al. (37) and Weikert et al. (21).

An iron deficiency anemia defined by decreased hemoglobin concentrations were found in 13% of women and 28% of men in the omnivorous group, 20/14% flexitarians, 21/17% vegetarians, and 29/18% vegans. Of the participants with anemia, reduced MCV values (<80 fl) were observed in 9% flexitarian women and one vegan (2%) woman. In men of this subgroup, MCV below the reference range was only detected in one vegetarian (6%).

Although the data on nutrient intake from self-reports showed a comparable iron intake on average 11–14 mg/d in the four studied diets, the iron status was worse in flexitarians, vegetarians, and vegans. This can be attributed to the low bioavailability of non-heme (approx. 3.7%) vs. heme iron (approx. 25%) (43). The absorption of non-heme iron varies strongly in dependence of dietary factors. Whereas phytic acid, calcium, polyphenols from coffee and tea reduce absorption of non-heme iron, simultaneously intake of ascorbic acid or other organic acids increase bioavailability of non-heme iron (43–45). While the dietary intake of vitamin C seems to be comparable between the four diets, the lower vitamin C concentrations measured in omnivores differ from the higher amounts in the vegetarian/vegan diets (*p* ≤ 0.05). This indicates for a higher intake of vitamin C and thereof an improvement of the bioavailability of non-heme iron (43).

Besides iron, iodine, selenium, and zinc are further critical trace elements because of their lower content in vegetarian/vegan diets and particularly their lower bioavailability from plants, due to the presence of e.g., phytic acid (44). Since it has been shown that urinary selenium is a reliable biomarker for assessing selenium status (46), our findings are in line with actual data on plasma concentrations of selenium and selenoproteins P in vegans vs. omnivores (21). The lower urinary zinc concentrations in flexitarians, vegetarians, and particularly vegans are in accordance with the markedly lower calculated dietary intake and thus indicate a poorer supply in comparison to omnivorous group. As zinc and vitamin A interact, the lowest level of urinary zinc in combination with the lowest average plasma concentrations of vitamin A in the vegan group point to an additional impairment of the physiological functions in the vegan diet (25). The average vitamin A concentrations were in line within the reference range, but the lowest concentrations in vegans differed significantly from the other groups. In twenty-one participants of the omnivorous group, fourteen flexitarians, nineteen vegetarians, and thirty-two vegans the vitamin A concentration fell below the reference range of 1.46 μmol/l. Evident from the dietary records, the fact that vegan diets are very low in vitamin A is partially compensated by the high intake of beta carotin in this group. A high intake of carotenoids is associated with a reduction of CVD risk (47). The lower urinary iodine levels in vegetarians, and vegans in comparison to omnivores are comparable to the data described by Weikert et al. (21). In the four diets under consideration, the mean values of iodine excretion are below the WHO cut-off values (<100 μg/l), indicating for an iodine deficiency. Thus, an adequate iodine intake by the diet must be promoted to avoid development of goiter (48).

## Conclusions

Most of the Europeans practice one of the before mentioned dietary patterns, each of which varies in their amount of animal-based products. The Academy of Nutrition and Dietetics postulates that adequately planned vegetarian diets are healthy, nutritionally adequate, and may provide health benefits in the prevention and treatment of non-communicable diseases (32). Overall, the data from the NuEva screening confirm this statement. The reduced consumption of animal products in the following order omnivores > flexitarians > vegetarians > vegans is associated with a decreased intake of energy, fat, and particularly SFA, cholesterol, disaccharides, and total sugar as well an increased intake of soluble and non-soluble dietary fibers, vitamin E, K, beta carotene, and manganese. In detail, the data suggests that flexitarian, vegetarian and vegan diets are nutrient-dense and could be recommended for weight management. The prevention of body weight gain and the observed blood lipid lowering effect of vegetarian and in particular vegan diets contribute to the prevention of CVD (49, 50).

However, the NuEva screening reveals an insufficient dietary intake of selenium, zinc, potassium, iron (women), calcium, vitamin B12, n-3 LC-PUFA, and vitamin D particularly in vegetarian and vegan diets.

### Recommendations

A regular consumption of the following foods can counteract the weak points detected in the NuEva screening (Table 7):

i) nuts, seeds, wheat bran and barley flakes as sources for zinc and selenium,
ii) paprika, pistachio, pumpkin seeds, cocoa etc. for optimal potassium intake,
iii) sesame seeds or tahini, cocoa, amaranth, cashews, pine nuts, and oats in combination with vitamin C or other organic acids to ensure an adequate iron intake,
iv) dairy products or plant-based foods enriched with vitamin B12, B12 supplements,
v) seaweeds (Nori) or microalgae oils, e.g., from schizochytrium sp. or ulkenia sp. for an optimal n-3 LC-PUFA status,
vi) adequate sun exposure is recommended to improve vitamin D status,
vii) supplementation of calcium and vitamin D and ensuring an adequate intake of high-quality protein to avoid an elevated fracture risk or the development of osteoporosis.

**Table 7:** Critical nutrients in vegetarian/vegan diets and suitable food sources.

**Critical nutrients and reference range for daily intake**	**Suitable food sources**	**Content per 100 g**	**Reference**
Zinc	Poppy seeds	7.9 mg	https://fdc.nal.usda.gov/fdc-app.html#/food-details/171330/nutrients
7–16 mg/d	Sesame seeds, dried	7.75 mg	https://fdc.nal.usda.gov/fdc-app.html#/food-details/170150/nutrients
	Wheat bran	7.26 mg	https://fdc.nal.usda.gov/fdc-app.html#/food-details/169722/nutrients
	Cashew, raw	5.78 mg	https://fdc.nal.usda.gov/fdc-app.html#/food-details/170162/nutrients
	Pecan nuts	4.53 mg	https://fdc.nal.usda.gov/fdc-app.html#/food-details/170182/nutrients
	Linseeds	4.34 mg	https://fdc.nal.usda.gov/fdc-app.html#/food-details/169414/nutrients
	Brazil nuts	4.06 mg	https://fdc.nal.usda.gov/fdc-app.html#/food-details/170569/nutrients
	Barley flakes	2.77 mg	https://fdc.nal.usda.gov/fdc-app.html#/food-details/170283/nutrients
	Pistachio	2.2 mg	https://fdc.nal.usda.gov/fdc-app.html#/food-details/170184/nutrients
Selenium	Brazil nuts	1920 μg	https://fdc.nal.usda.gov/fdc-app.html#/food-details/170569/nutrients
60–70 μg/d	Wheat bran	77.6 μg	https://fdc.nal.usda.gov/fdc-app.html#/food-details/169722/nutrients
	Barley flakes	37.7 μg	https://fdc.nal.usda.gov/fdc-app.html#/food-details/170283/nutrients
	Sesame seeds, dried	34.4 mg	https://fdc.nal.usda.gov/fdc-app.html#/food-details/170150/nutrients
	Cheese, parmesan, grated	34.4 mg	https://fdc.nal.usda.gov/fdc-app.html#/food-details/171247/nutrients
	Linseeds	25.4 μg	https://fdc.nal.usda.gov/fdc-app.html#/food-details/169414/nutrients
	Cashew, raw	19.9 mg	https://fdc.nal.usda.gov/fdc-app.html#/food-details/170162/nutrients
	Poppy seeds	13.5 μg	https://fdc.nal.usda.gov/fdc-app.html#/food-details/171330/nutrients
	Pecan nuts	3.8 μg	https://fdc.nal.usda.gov/fdc-app.html#/food-details/170182/nutrients
Potassium	Paprika	2280 mg	https://fdc.nal.usda.gov/fdc-app.html#/food-details/171329/nutrients
4000 μg/d	Cocoa	1520 mg	https://fdc.nal.usda.gov/fdc-app.html#/food-details/169593/nutrients
	Pistachio	1020 mg	https://fdc.nal.usda.gov/fdc-app.html#/food-details/170184/nutrients
	Pumpkin seeds	919 mg	https://fdc.nal.usda.gov/fdc-app.html#/food-details/170188/nutrients
	Almonds	733 mg	https://fdc.nal.usda.gov/fdc-app.html#/food-details/170567/nutrients
	Potatoes	417 mg	https://fdc.nal.usda.gov/fdc-app.html#/food-details/170027/nutrients
	Lentils, mature seeds, cooked, boiled	369 mg	https://fdc.nal.usda.gov/fdc-app.html#/food-details/172421/nutrients
	Carrots	320 mg	https://fdc.nal.usda.gov/fdc-app.html#/food-details/170393/nutrients
	Kohlrabi	320 mg	https://fdc.nal.usda.gov/fdc-app.html#/food-details/168424/nutrients
	Mushrooms	318 mg	https://fdc.nal.usda.gov/fdc-app.html#/food-details/169251/nutrients
	Lettuce	247 mg	https://fdc.nal.usda.gov/fdc-app.html#/food-details/169247/nutrients
	Cabbage	170 mg	https://fdc.nal.usda.gov/fdc-app.html#/food-details/169975/nutrients
Iron^*^	Sesame seeds, dried	14.6 mg	https://fdc.nal.usda.gov/fdc-app.html#/food-details/170150/nutrients
10–15 mg/d	Cocoa	13.9 mg	https://fdc.nal.usda.gov/fdc-app.html#/food-details/169593/nutrients
	Sesame tahini	9 mg	https://fdc.nal.usda.gov/fdc-app.html#/food-details/446287/nutrients
	Amaranth, grain, uncooked	7.61 mg	https://fdc.nal.usda.gov/fdc-app.html#/food-details/170682/nutrients
	Cashew, raw	6.68 mg	https://fdc.nal.usda.gov/fdc-app.html#/food-details/170162/nutrients
	Pine nuts	5.53 mg	https://fdc.nal.usda.gov/fdc-app.html#/food-details/170591/nutrients
	Quinoa, uncooked	4.57 mg	https://fdc.nal.usda.gov/fdc-app.html#/food-details/168874/nutrients
	Oats	4.5 mg	https://fdc.nal.usda.gov/fdc-app.html#/food-details/368739/nutrients
	Chickpeas	2.89 mg	https://fdc.nal.usda.gov/fdc-app.html#/food-details/173757/nutrients
	Spinach, raw	2.71 mg	https://fdc.nal.usda.gov/fdc-app.html#/food-details/168462/nutrients
Calcium	Sesame seeds, dried	975 mg	https://fdc.nal.usda.gov/fdc-app.html#/food-details/170150/nutrients
1,000 mg/d	Cheese, parmesan, grated	853 mg	https://fdc.nal.usda.gov/fdc-app.html#/food-details/171247/nutrients
	Almonds	269 mg	https://fdc.nal.usda.gov/fdc-app.html#/food-details/170567/nutrients
	Spinach, raw	99 mg	https://fdc.nal.usda.gov/fdc-app.html#/food-details/168462/nutrients
	Broccoli	47 mg	https://fdc.nal.usda.gov/fdc-app.html#/food-details/170379/nutrients
	Fortified foods, such as oat milk		
Vitamin B12	Cheese, swiss	3.02 μg	https://fdc.nal.usda.gov/fdc-app.html#/food-details/746767/nutrients
4 μg/d	Cheese, mozzarella, low moisture, part-skim	1.65 μg	https://fdc.nal.usda.gov/fdc-app.html#/food-details/329370/nutrients
	Cheese, parmesan, grated	1.35 μg	https://fdc.nal.usda.gov/fdc-app.html#/food-details/325036/nutrients
	Cheese, cheddar	1.06 μg	https://fdc.nal.usda.gov/fdc-app.html#/food-details/328637/nutrients
	Cheese, ricotta, whole milk	0.78 μg	https://fdc.nal.usda.gov/fdc-app.html#/food-details/746766/nutrients
	Yogurt, plain, low fat	0.56 μg	https://fdc.nal.usda.gov/fdc-app.html#/food-details/170886/nutrients
	Milk, whole	0.54 μg	https://fdc.nal.usda.gov/fdc-app.html#/food-details/1097512/nutrients
	Plant-based foods enriched with vitamin B12		
	B12 supplements (chewable tablets, drops, toothpaste etc.)		
Long-chain n-3 PUFA 250–500 mg/d	Nori (seaweeds)	80 mg	https://fdc.nal.usda.gov/fdc-app.html#/food-details/168458/nutrients
	Supplements, capsules, or microalgae oils, e.g., from schizochytrium sp., ulkenia sp.		
Vitamin D 20 μg/d	Adequate sun exposure or supplementation		

* *Combination of plant-based foods with sources of vitamin C or other organic acids and avoiding the simultaneous consumption of foods and beverages containing polyphenols (tea, coffee), phytic acid to increase iron bioavailability*.

Future R&D activities should focus on the improvement of nutrient profiles of traditional plant food, e.g., by optimal variety selection of seeds, improvement of the soil quality, and reduction in processing steps of plant-based foods to avoid loss of their valuable nutrients.

In summary, the NuEva study highlights the need for development and distribution of practical nutritional concepts adapted to individual dietary preferences to ensure an adequate nutrient intake and to avoid deficiency symptoms and risk of associated disorders for all diets under consideration.

### Strengths and Limitations

The NuEva study is designed to identify critical nutrients relating to the implementation of one of the studied diets (omnivores, flexitarians, vegetarians, vegans,) by evaluation of a self-reported dietary protocol and measured parameters reflecting nutrient status in serum, plasma, and 24h urine. Due to a comprehensive assessment of biomarkers and data on body composition, the NuEva study allows evaluating physiological benefits or possible physiological consequences resulting from the implementation of the studied nutritional habits with focus on cardiovascular risk.

A one-time self-reported dietary protocol over a defined period is one of the limitations of the NuEva screening. Self-reports on dietary intake bear the risk of over-reporting of healthy foods and under-reporting of high-energy, low nutrient foods. The dietary protocols were calculated with the software PRODI^®^ version 6.4 (Nutri-Science, Stuttgart, Germany) for professional dietary counseling and therapy. The calculations on nutrients composition based on the “Bundeslebensmittelschlüssel” and further nutrition tables. In this context, differences between nutrient profiles calculated with the software and the nutrient composition of the consumed foods, adding to the limitations encountered in this study. Variations of nutrient profiles can depend on types, preparation conditions and feeding conditions.

A further limitation is the generalizability of the data which represent a regional sample as the participants were recruited from central East Germany.

The calculated nutrient intake form self-reports was related to the recommendations for nutrient intake from the German Society of Nutrition (DGE e.V.). Currently, these guidelines do not consider the implementation of diets differing in their proportion of animal-based foods.

## Data Availability Statement

The datasets presented in this article are not readily available because of an ongoing evaluation of the datasets by the study team. Requests to access the datasets should be directed to the corresponding author.

## Ethics Statement

The studies involving human participants were reviewed and approved by Ethical Committee of the Friedrich Schiller University of Jena. The patients/participants provided their written informed consent to participate in this study.

## Author Contributions

CD was responsible for acquisition of funding, conceptualization and trial design, conduction of the NuEva study, data acquisition, and she wrote the original draft. As study physician KD was responsible for medical care of the NuEva participants during the study. TW performed the statistical analysis of the data sets based on a constituted statistical plan. PS and CD supervised the statistical analysis. CR supported the data acquisition and sample management. MK was responsible for data acquisition and critical revision of the manuscript. All authors contributed to the article and approved the submitted version.

## Funding

This research was funded by the German Federal Ministry of Research and Education (Grant Number: 01EA1708). The funders had no role in the design of the study; in the collection, analyses, or interpretation of data; in the writing of the manuscript, or in the decision to publish the results.

## Conflict of Interest

The authors declare that the research was conducted in the absence of any commercial or financial relationships that could be construed as a potential conflict of interest.

## Publisher's Note

All claims expressed in this article are solely those of the authors and do not necessarily represent those of their affiliated organizations, or those of the publisher, the editors and the reviewers. Any product that may be evaluated in this article, or claim that may be made by its manufacturer, is not guaranteed or endorsed by the publisher.
