# Corrigendum: Nutrient intake and nutrition status in vegetarians and vegans in comparison to omnivores—the nutritional evaluation (NuEva) study

**DOI:** 10.3389/fnut.2022.975159

**Published:** 2022-07-27

**Authors:** Christine Dawczynski, Thomas Weidauer, Cora Richert, Peter Schlattmann, Kristin Dawczynski, Michael Kiehntopf

**Affiliations:** ^1^Junior Research Group Nutritional Concepts, Institute of Nutritional Sciences, Friedrich Schiller University, Jena, Germany; ^2^Competence Cluster for Nutrition and Cardiovascular Health (nutriCARD), Halle-Jena-Leipzig, Leipzig, Germany; ^3^Institute of Clinical Chemistry and Laboratory Diagnostics, University Hospital, Jena, Germany; ^4^Department of Medical Statistics, Informatics and Data Science, University Hospital, Jena, Germany; ^5^Department for Pediatrics and Adolescent Medicine, Sophien- and Hufeland Hospital, Weimar, Germany

**Keywords:** vegans, vegetarians, omnivores, nutrient intake, blood lipids, body weight

In the published article, there was an error in the legend for “**Table 1**. Characteristics of the study collective - NuEva-screening (Median/Interquartile range (IQR); (Min - Max)).” as published. The Information “^*^Diet groups with different indices differ significantly (*p* < 0.05)” was lost. The corrected legend appears below.

“Groups: 1 = omnivores, 2 = flexitarians, 3 = vegetarians, 4 = vegans.

^*^Diet groups with different indices differ significantly (*p* < 0.05).”

In the published article, there was an error in the legend for “**Table 2**. Daily intake of energy and macronutrients (self-reports, 5 days) - NuEva-screening (Median/IQR; (Min - Max)).” as published. The Information “^*^Diet groups with different indices differ significantly (*p* < 0.05)” was lost. The corrected legend appears below.

“Groups: 1 = omnivores, 2 = flexitarians, 3 = vegetarians, 4 = vegans.

Adjusted for age: Σ monounsaturated fatty acids (%).

^§^Reference intake: DGE, 2019.

^*^Diet groups with different indices differ significantly (*p* < 0.05).”

In the published article, there was an error in the legend for “**Table 3**. Daily intake of vitamins (self-reports, 5 days) - NuEva-screening (Median / IQR; (Min - Max)).” as published. The Information “^*^Diet groups with different indices differ significantly (*p* < 0.05)” was lost. The corrected legend appears below.

“Groups: 1 = omnivores, 2 = flexitarians, 3 = vegetarians, 4 = vegans.

^§^Reference intake: DGE, 2019.

Significant influence of sex: vitamin B1, B2, B12.

^*^Diet groups with different indices differ significantly (*p* < 0.05).”

In the published article, there was an error in the legend for “**Table 4**. Daily intake of minerals and trace elements (self-reports, 5 days) - NuEva-screening (Median / IQR; (Min - Max)).” as published. The Information “^*^Diet groups with different indices differ significantly (*p* < 0.05)” was lost. To complete the data, we would like to insert the information that the calculation of iodine and selenium intake was not possible. The corrected legend appears below.

“Groups: 1 = omnivores, 2 = flexitarians, 3 = vegetarians, 4 = vegans.

Adjusted for BMI: Iodine (μg).

^§^Reference intake: DGE, 2019.

Significant influence of sex: chloride, iron, copper, zinc.

The selenium intake was not calculated because the nutritional software (PRODI^®^) does not provide any information on the selenium levels in foods.

The iodine intake was not calculated because the additional intake by fortified table salt was unknown.

^*^Diet groups with different indices differ significantly (*p* < 0.05).”

In the published article, there was an error in the legend for “**Table 5**. Anthropometric data, body composition and blood lipids – NuEva-screening (Median / IQR; (Min - Max)).” as published. The Information “^*^Diet groups with different indices differ significantly (*p* < 0.05)” was lost. The corrected legend appears below.

“Adjusted for age: BMI, total cholesterol, LDL cholesterol, apolipoprotein A1, apolipoprotein B.

Adjusted for BMI: waist circumferences.

Significant influence of sex: weight, BMI, body cell mass, extracellular mass, BCM/ECM, metabolic rate, body fat, body water, lean body mass, phase angle, cell amount, HDL cholesterol, apolipoprotein A1/ apolipoprotein B.

^*^Diet groups with different indices differ significantly (*p* < 0.05).”

In the published article, there was an error in the legend for “Table 6. Vitamins, minerals and trace elements in plasma/serum and 24h urine – NuEva-screening (Median / IQR; (Min - Max)).” as published. The Information “^*^Diet groups with different indices differ significantly (*p* < 0.05)” was lost. In addition, the information on 4cB12score [^§^4cB12 score - combined index of B12 deficiency (normal range:−0.5 - 1.0)] was also lost. The corrected legend appears below.

**Table 6:** Vitamins, minerals and trace elements in plasma/serum and 24h urine – NuEva-screening (Median / IQR; (Min - Max)).

		**Group 1**				**Group 2**				**Group 3**				**Group 4**			
**Parameter**	**Sex**	**Median**	**/**	**IQR**	***p***	**Median**	**/**	**IQR**	***p***	**Median**	**/**	**IQR**	***p***	**Median**	**/**	**IQR**	***p***
Plasma / serum																	
**Biotin**	All	249	/	108	a	305	/	161	b	284	/	136	a,b	291	/	166	b
(ng/l)		(94–1,000)				(143–1,000)				(62–1,000)				(101–1,000)			
**Folate**	All	7.20	/	6.00	a,b	8.65	/	4.18	a,b	8.10	/	3.90	a	10.40	/	5.03	b
(μg/l)		(2.2–16.9)				(3.2–16.5)		(2.9–16.9)		(3.7–18.3)							
**Vitamin B_12_**	All	242	/	94	a	246	/	119	a	208	/	110	b	213	/	161	a,b
(pmol/l)		(109–567)				(116–508)				(110–966)				(128–712)			
**Holo-Transcobalamine**	All	80.8	/	44.1	a	73.9	/	35.1	a	54.9	/	29.8	b	54.9	/	47.6	c
(pmol/l)		(39–227)				(26–180)				(11–356)				(14–327)			
**Homocysteine**	All	9.5	/	4.4	a	10.5	/	4.1	a	10.2	/	4.4	a	10.0	/	3.7	a
(μmol/l)		(4.4–21.2)				(5.3–19.2)				(5.2–33.5)				(3.7–37.8)			
**Methyl malonic acid**	All	17.0	/	8.5	a	20.0	/	10.0	a	21.0	/	13.0	a	18.5	/	12.3	a
(μg/l)		(9–65)				(8–57)				(9–82)				(7–64)			
**4cB12 score^§^**	All	0.34	/	0.58	a	0.24	/	0.52	a,c	0.02	/	0.75	c	0.08	/	0.89	b,c
		(−0.51 to 1.33)				(−0.66 to 1.45)				(−2.05 to 2.07)				(−1.44 to 1.52)			
**Vitamin B_1_**	All	137.2	/	34.2	a,b	140.0	/	37.6	a	130.3	/	37.6	b	133.0	/	33.3	a,b
(nmol/l)		(79 – 235)				(72–215)				(63–275)				(91–208)			
**Vitamin B_2_**	All	230	/	54.3	a	247	/	37.0	b	225	/	56.0	a,c	220	/	44.5	a,c
(μg/l)		(150–334)				(175–343)				(155–335)				(147–318)			
**Vitamin B_6_**	All	51.7	/	40.8	a	54.6	/	28.6	a	48.7	/	29.1	a	54.8	/	30.8	a
(nmol/l)		(20–264)				(18–187)				(14–257)				(15–194)			
**Vitamin C**	All	6.9	/	3.7	a	7.8	/	5.8	a,b	8.8	/	4.7	b	10.4	/	4.1	c
(mg/l)		(0.4–13.1)				(1.6–19.5)				(0.6–16.6)				(3.0–20.4)			
**Vitamin A**	All	1.61	/	0.62	a	1.75	/	0.58	a	1.67	/	0.59	a	1.35	/	0.42	b
(μmol/l)		(0.9–3.1)				(1.0–3.0)				(1.0–2.9)				(0.9–2.9)			
**Vitamin D**	All	70.7	/	21.6	a	65.4	/	26.6	a	68.3	/	34.3	a	65.0	/	22.3	a
(nmol/l)		(17–134)				(34–118)				(18–145)				(16–181)			
**Vitamin E**	All	26.7	/	8.9	a	27.1	/	7.8	a	25.0	/	7.3	a,b	24.0	/	6.8	b
(μmol/l)		(17–72)				(17–60)				(14–44)				(13–47)			
**Ferritin**	All	80.1	/	89.6	a	31.3	/	44.2	b	31.2	/	19.6	b	29.9	/	39.8	b
(μg/l)		(3.1–455)				(2.5–223)				(4.5–267)				(1.5–169)			
**Transferrin**	All	2.5	/	0.5	a	2.8	/	0.78	b	2.8	/	0.5	b	2.8	/	0.5	b
(g/l)		(2.0–3.9)				(1.9–4.7)				(2.0–3.9)				(1.8–4.1)			
**Transferrin saturation**	All	28.5	/	13.2	a	26.2	/	18.6	a	27.0	/	13.3	a	30.9	/	20.1	a
(%)		(6.4–88.0)				(2.9–57.7)				(6.6–60.0)				(7.8–73.0)			
**24h urine**																	
**Magnesium**	All	4.30	/	2.10	a	4.40	/	1.93	a	4.80	/	1.60	a	4.90	/	2.20	a
(mmol/24h)		(1.0–10.6)				(1.4–9.5)				(1.0–8.7)				(1.3–9.9)			
**Sodium**	All	143	/	79	a	113	/	71	a	146	/	80	a	128	/	88	a
(mmol/24h)		(61–291)				(40–299)				(48–282)				(42–346)			
**Selenium**	All	0.25	/	0.19	a	0.19	/	0.13	b	0.20	/	0.09	b	0.16	/	0.12	b
(μmol/ 24h)		(0.07–0.77)				(0.06–0.76)				(0.07–0.66)				(0.06–0.91)			
**Zinc**	m	10.75	/	3.33	a	8.30	/	8.00	a	8.25	/	4.53	a	6.05	/	3.55	a
(μmol/24h)		(3.6–32.8)				(3.4–19.7)				(2.8–13.6)				(4.3–13.4)			
	w	5.85	/	4.23	a	5.20	/	3.08	a	5.60	/	4.20	a	4.20	/	2.70	b
		(3.2–27.2)				(1.8–14.6)				(1.7–18)				(0.8–9.5)			
	All	7.85	/	5.58	a	5.50	/	4.60	b,c	6.10	/	3.90	b	5.00	/	3.30	c
		(3.2–32.8)				(1.8–19.7)				(1.7–18)				(0.8–13.4)			
**Iodine**	All	53.0	/	47.5	a	52.0	/	35.5	a,b	42.0	/	27.0	a,b	21.5	/	16.8	b
(μg/l)		(17–268)				(13–192)				(6–335)				(8–509)			

Significant influence of sex: zinc.

Adjusted for age: vitamin E.

*Diet groups with different indices differ significantly (p < 0.05).

§ 4cB12 score–combined index of B12 deficiency (normal range:−0.5 - 1.0).

“Significant influence of sex: zinc.

Adjusted for age: vitamin E.

^*^Diet groups with different indices differ significantly (*p* < 0.05).

^§^4cB12 score - combined index of B12 deficiency (normal range:−0.5 - 1.0).”

In the published article, there was an error in “Table 6. Vitamins, minerals and trace elements in plasma/serum and 24h urine – NuEva-screening (Median / IQR; (Min - Max)).” as published. The units for ferritin (μg/l), transferrin (g/l) and transferrin saturation (%) were lost in Table 6. The corrected “Table 6. Vitamins, minerals and trace elements in plasma/serum and 24h urine – NuEva-screening (Median / IQR; (Min - Max)).” and its legend appear below.

The authors apologize for this error and state that this does not change the scientific conclusions of the article in any way. The original article has been updated.

## Publisher's note

All claims expressed in this article are solely those of the authors and do not necessarily represent those of their affiliated organizations, or those of the publisher, the editors and the reviewers. Any product that may be evaluated in this article, or claim that may be made by its manufacturer, is not guaranteed or endorsed by the publisher.

