# Supplementary material for: Nutrient Intake and Nutrition Status in Vegetarians and Vegans in Comparison to Omnivores - the Nutritional Evaluation (NuEva) Study
Source: Front Nutr. 2022 May 16;9:819106. doi: 10.3389/fnut.2022.819106 (PMC9149309; doi:10.3389/fnut.2022.819106)
Supplement: Supplementary file 1 [file Data_Sheet_1.PDF]

Supplemental data

Table S1: Biochemical methods and reference ranges

Parameter	Method/Instrument	Reference range	Institute/ Reference
Plasma, serum			
Total cholesterol, (mmol/l)	Abbott Architect CI 16200 analyzer (Abbott, Wiesbaden, Germany)	< 5,2	Institute of Clinical Chemistry and Laboratory Diagnostics, University Hospital Jena, accredited reference laboratory (D-ML-13144-04, valid until 28.06.2023)
LDL cholesterol, (mmol/l)	Abbott Architect CI 16200 analyzer (Abbott, Wiesbaden, Germany)	< 3.35	Institute of Clinical Chemistry and Laboratory Diagnostics, University Hospital Jena, accredited reference laboratory (D-ML-13144-04, valid until 28.06.2023)
HDL cholesterol, (mmol/l)	Abbott Architect CI 16200 analyzer (Abbott, Wiesbaden, Germany)	> 1.03	Institute of Clinical Chemistry and Laboratory Diagnostics, University Hospital Jena, accredited reference laboratory (D-ML-13144-04, valid until 28.06.2023)
Triglycerides (mmol/l)	Abbott Architect CI 16200 analyzer (Abbott, Wiesbaden, Germany)	< 1.7	Institute of Clinical Chemistry and Laboratory Diagnostics, University Hospital Jena, accredited reference laboratory (D-ML-13144-04, valid until 28.06.2023)
Malondialdehyde-modified LDL (U/l)	Enzyme-linked Immunosorbent Assay (ELISA)	< 110 U/l	Dianovis GmbH / ImmBiomed GmbH & Co. KG (Product no. 447839)
Apolipoprotein A (g/l)	COBAS INTEGRA 400 plus System	female: 1.08 - 2.25 male: 1.04 - 2.02	Dianovis GmbH
Apolipoprotein B (g/l)	COBAS INTEGRA 400 plus System	female: 0.60 - 1.17 male: 0.66 - 1.33	Dianovis GmbH
high-sensitive c-reactive protein (mg/dl)	Abbott Architect CI 16200 analyzer (Abbott, Wiesbaden, Germany)	< 0.3	Institute of Clinical Chemistry and Laboratory Diagnostics, University Hospital Jena, accredited reference laboratory (D-ML-13144-04, valid until 28.06.2023)
Biotin (ng/l)	Enzyme-linked Immunosorbent Assay (ELISA)	> 250	Dianovis GmbH / IDK® Biotin ELISA for the in vitro determination of biotin (vitamin H) in serum, plasma, urine and milk. ImmunDiagnostic manual. Immundiagnostik AG, 2019, REF K8141
Vitamin B12 (pmol/l)	Abbott Architect CI 16200 analyzer (Abbott, Wiesbaden, Germany)	197 - 7712	Institute of Clinical Chemistry and Laboratory Diagnostics, University Hospital Jena, accredited reference laboratory (D-ML-13144-04, valid until 28.06.2023)
Folate (µg/l)	Abbott Architect CI 16200 analyzer (Abbott, Wiesbaden, Germany)	3.9 - 26.8 Weikert et al. 2020	Institute of Clinical Chemistry and Laboratory Diagnostics, University Hospital Jena, accredited reference laboratory (D-ML-13144-04, valid until 28.06.2023
Holo-trans-cobalamin (pmol/l)	Abbott Architect CI 16200 analyzer (Abbott, Wiesbaden, Germany)	> 37.52	Institute of Clinical Chemistry and Laboratory Diagnostics, University Hospital Jena, accredited reference laboratory (D-ML-13144-04, valid until 28.06.2023)

Homo-<br>cysteine<br>(µmol/l)	High-performance liquid chromatography<br>(HPLC; Shimadzu®)	5 ≤ 15<br>Weikert et al. 2020	Institute of Clinical Chemistry and Laboratory Diagnostics, University Hospital Jena, accredited reference laboratory (D-ML-13144-04, valid until 28.06.2023)
Methyl malonic acid<br>(µg/l)	Liquid chromatography–mass spectrometry/ mass spectrometry (LC-MS/MS)	8.6 ≤ 32.0	Dianovis GmbH /<br>LC-MS/MS, Chromsystems Instruments & Chemicals GmbH, DIN EN ISO 9001, DIN EN ISO 13485, ISO 13485 CMDR
4cB12 score = a combined indicator of vitamin B12 status	Calculation	Normal range:<br>-0.5 to 1.0<br>Low vitamin B12 supply:<br>-1.5 to -0.5<br>Potential B12 deficiency:<br>-1.5 to -2.5	The 4cB12 score was calculated from the following parameters: Holo-transcobalamin, vitamin B12, methyl malonic acid, homocysteine [13]
Vitamin B1<br>(nmol/l)	HPLC	47 - 1412	Institute of Clinical Chemistry and Laboratory Diagnostics, University Hospital Jena, accredited reference laboratory (D-ML-13144-04, valid until 28.06.2023) / Chromsystems & Chemicals GmbH, DIN EN ISO 9001:2008 & DIN EN ISO 13485:2007-10
Vitamin B2<br>(µg/l)	HPLC	180 - 295	Dianovis GmbH /<br>ClinRep® HPLC Komplettkit, RECIPE, REF 25000
Vitamin B6<br>(nmol/l)	HPLC	14.6 - 72.8	Institute of Clinical Chemistry and Laboratory Diagnostics, University Hospital Jena, accredited reference laboratory (D-ML-13144-04, valid until 28.06.2023) / Chromsystems & Chemicals GmbH, DIN EN ISO 9001:2008 & DIN EN ISO 13485:2007-10
Vitamin C<br>(mg/l)	HPLC	4 - 15	Dianovis GmbH /<br>ClinRep® HPLC Komplettkit, RECIPE, REF 28000
Vitamin A<br>(µmol/l)	HPLC (Shimadzu®)	1.46 - 2.85<br>Weikert et al. 2020	Institute of Clinical Chemistry and Laboratory Diagnostics, University Hospital Jena, accredited reference laboratory (D-ML-13144-04, valid until 28.06.2023)
Vitamin D<br>(25(OH)D)<br>(nmol/l)	Abbott Architect CI 16200 analyzer (Abbott, Wiesbaden, Germany)	> 75	Institute of Clinical Chemistry and Laboratory Diagnostics, University Hospital Jena, accredited reference laboratory (D-ML-13144-04, valid until 28.06.2023)
Vitamin E<br>(µmol/l)	HPLC (Shimadzu®)	11.6 - 46.4	Institute of Clinical Chemistry and Laboratory Diagnostics, University Hospital Jena, accredited reference laboratory (D-ML-13144-04, valid until 28.06.2023)
Calcium<br>(mmol/l)	Abbott Architect CI 16200 analyzer (Abbott, Wiesbaden, Germany)	2.15 - 2.50	Institute of Clinical Chemistry and Laboratory Diagnostics, University Hospital Jena, accredited reference laboratory (D-ML-13144-04, valid until 28.06.2023)
Potassium<br>(mmol/l)	Abbott Architect CI 16200 analyzer (Abbott, Wiesbaden, Germany)	3.4 - 4.5	Institute of Clinical Chemistry and Laboratory Diagnostics, University Hospital Jena, accredited reference laboratory (D-ML-13144-04, valid until 28.06.2023)
Iron	Abbott Architect CI 16200	Male: 4.1 -	Institute of Clinical Chemistry and Labora-

(µmol/l)	analyzer (Abbott, Wiesbaden, Germany)	30.1<br>Female: 4.1 - 26.7	tory Diagnostics, University Hospital Jena, accredited reference laboratory (D-ML-13144-04, valid until 28.06.2023)
Ferritin<br>(µg/l)	Abbott Architect CI 16200 analyzer (Abbott, Wiesbaden, Germany)	Male: 30 - 400<br>Female: 13 - 150	Institute of Clinical Chemistry and Laboratory Diagnostics, University Hospital Jena, accredited reference laboratory (D-ML-13144-04, valid until 28.06.2023)
Transferrin<br>(g/l)	Abbott Architect CI 16200 analyzer (Abbott, Wiesbaden, Germany)	2.0 - 3.6	Institute of Clinical Chemistry and Laboratory Diagnostics, University Hospital Jena, accredited reference laboratory (D-ML-13144-04, valid until 28.06.2023)
Hemoglobin<br>(mmol/l)	XN 1000 (Sysmex®)	7.6 - 9.5	Institute of Clinical Chemistry and Laboratory Diagnostics, University Hospital Jena, accredited reference laboratory (D-ML-13144-04, valid until 28.06.2023)
Mean corpuscular volume, (MCV) (fl)	XN 1000 (Sysmex®)	80 - 96	Institute of Clinical Chemistry and Laboratory Diagnostics, University Hospital Jena, accredited reference laboratory (D-ML-13144-04, valid until 28.06.2023)
24h urine			
Magnesium<br>(mmol/24h)	Abbott Architect CI 16200 analyzer (Abbott, Wiesbaden, Germany)	No information available	Institute of Clinical Chemistry and Laboratory Diagnostics, University Hospital Jena, accredited reference laboratory (D-ML-13144-04, valid until 28.06.2023)
Sodium<br>(mmol/24h)	Abbott Architect CI 16200 analyzer (Abbott, Wiesbaden, Germany)	94 - 222	Institute of Clinical Chemistry and Laboratory Diagnostics, University Hospital Jena, accredited reference laboratory (D-ML-13144-04, valid until 28.06.2023)
Selenium<br>(µmol/ 24h)	Atomic absorption spectroscopy (AAS) ZEEnit 60 (Analytik Jena AG, Jena, Germany)	No information available	Institute of Clinical Chemistry and Laboratory Diagnostics, University Hospital Jena, accredited reference laboratory (D-ML-13144-04, valid until 28.06.2023)
Zinc<br>(µmol/24h)	AAS 5 FL (Analytik Jena AG, Jena, Germany)	No information available	Institute of Clinical Chemistry and Laboratory Diagnostics, University Hospital Jena, accredited reference laboratory (D-ML-13144-04, valid until 28.06.2023)
Iodine<br>(µl/l)	Inductively-coupled-plasma mass-spectrometry (ICP-MS)	100 - 200	Dianovis GmbH /<br>Instrument: Nexion 300 D Perkin Elmer
Albumin<br>(mg/l)	Abbott Architect CI 16200 analyzer (Abbott, Wiesbaden, Germany)	No information available	Institute of Clinical Chemistry and Laboratory Diagnostics, University Hospital Jena, accredited reference laboratory (D-ML-13144-04, valid until 28.06.2023)
Creatinine<br>(mmol/l)	Abbott Architect CI 16200 analyzer (Abbott, Wiesbaden, Germany)	8.0 - 26.5	Institute of Clinical Chemistry and Laboratory Diagnostics, University Hospital Jena, accredited reference laboratory (D-ML-13144-04, valid until 28.06.2023)

Table S2a: Socio-economic status - NuEva-screening.

Socio-economic status		Group 1	Group 2	Group 3	Group 4	P value
married, living together	**Marital status**	21	18	12	8	0.053
married, living separately		0	2	2	0	
divorced		3	2	2	3	
not married, living together with partner		17	12	20	9	
not married, partner lives separately		2	11	6	10	
single		18	23	21	27	
single parent		4	2	2	0	
not specified		0	0	0	1	
primary school leaving certificate	**Graduation**	0	0	1	0	0.002
secondary school leaving certificate		17	7	3	2	
university entrance qualification		48	62	59	56	
finished school without degree		0	0	0	0	
not specified		0	1	2	0	
yes	**Completed vocational training**	33	29	18	18	0.024
no		31	32	41	39	
not specified		1	9	6	1	
yes	**University degree**	35	35	34	22	0.210
no		27	32	29	36	
not specified		3	3	2	0	
1	**Number of persons living in the household**	12	15	15	12	0.832
2		18	26	18	20	
3		17	14	19	12	
4		13	11	8	8	
5		1	2	3	3	
6		2	1	1	1	
7		0	0	0	1	
not specified		2	1	1	1	
< 500€	**Household net income (per month)**	2	11	4	8	0.000
501 - 800€		8	9	17	14	
801 - 1000€		0	4	3	7	
1001 - 1500€		4	10	5	9	
1501 - 2000€		7	6	6	2	
2001 - 2500€		12	7	12	4	
2501 - 3000€		5	4	2	3	
> 3000€		26	16	13	9	
not specified		1	3	3	2	

Groups: 1 = omnivores, 2 = flexitarians, 3 = vegetarians, 4 = vegans
NS not significant

Table S2b: Supplement intake - NuEva-screening.

Supplement intake		Group 1	Group 2	Group 3	Group 4	P value
yes	**Nutritional supplements**	19	28	26	48	0.000
no		46	40	39	10	
not specified		0	2	0	0	
Vitamin D + Vitamin K	**Nutritional supplements**	6	8	8	14	0.118
Vitamin E		0	2	1	1	0.184
Fish oil		3	1	4	2	0.643
Vitamin B_6		0	0	1	1	0.232
Biotin		0	2	1	0	0.166
Folic acid		0	4	1	3	0.099
Vitamin B_{12}		1	1	11	40	0.000
Calcium		2	4	0	1	0.375
Zinc		1	5	2	4	0.060
Magnesium		3	2	4	3	0.515
Iron		3	2	3	5	0.045
Selenium		0	1	2	2	0.245
Iodine		1	2	0	2	0.057
yes	**Food allergy**	14	19	16	17	0.961
no		51	51	49	41	
Smoker	**Smoking status**	8	7	9	4	0.587
Ex-Smoker		12	9	13	13	0.800

Groups: 1 = omnivores, 2 = flexitarians, 3 = vegetarians, 4 = vegans
NS not significant
